# Gut Microbiota in Military International Travelers with Doxycycline Malaria Prophylaxis: Towards the Risk of a Simpson Paradox in the Human Microbiome Field

**DOI:** 10.3390/pathogens10081063

**Published:** 2021-08-21

**Authors:** Emilie Javelle, Aurélie Mayet, Matthieu Million, Anthony Levasseur, Rodrigue S. Allodji, Catherine Marimoutou, Chrystel Lavagna, Jérôme Desplans, Pierre Edouard Fournier, Didier Raoult, Gaëtan Texier

**Affiliations:** 1 Laveran Military Teaching Hospital, Boulevard Alphonse Laveran, 13013 Marseille, France; 2 IRD, AP-HM, SSA, VITROME, Aix Marseille University, 13000 Marseille, France; Pierre-edouard.fournier@univ-amu.fr (P.E.F.); Gaetan.TEXIER@univ-amu.fr (G.T.); 3 IHU-Méditerranée Infection, 19–21 Boulevard Alphonse Laveran, 13013 Marseille, France; Matthieu.MILLION@ap-hm.fr (M.M.); anthony.levasseur@univ-amu.fr (A.L.); Didier.raoult@ap-hm.fr (D.R.); 4 Centre d’Epidémiologie et de Santé Publique des Armées (CESPA), 13014 Marseille, France; aurelie.mayet@intradef.gouv.fr (A.M.); catherine.marimoutou@chu-reunion.fr (C.M.); chrystel.lavagna-sevenier@intradef.gouv.fr (C.L.); jerome.desplans@intradef.gouv.fr (J.D.); 5 INSERM, IRD, SESSTIM, Sciences Economiques & Sociales de la Santé & Traitement de l’Information Médicale, Aix Marseille University, 13000 Marseille, France; 6 IRD, AP-HM, SSA, MEPHI, Aix Marseille University, 13000 Marseille, France; 7 Radiation Epidemiology Team, CESP, Inserm U1018, 94800 Villejuif, France; Rodrigue.ALLODJI@gustaveroussy.fr; 8 Université Paris-Saclay, UVSQ, Inserm, CESP, 94807 Villejuif, France; 9 Department of Research, Gustave Roussy, 94800 Villejuif, France; 10 CIC Inserm 1410, CHU de La Réunion, 97400 La Réunion, France

**Keywords:** gut microbiota, tetracycline, doxycycline, malaria prophylaxis, travel, soldiers, mission, deployment, group-effect, Simpson paradox, *Bacteroides*, *Rothia*

## Abstract

Dysbiosis, developed upon antibiotic administration, results in loss of diversity and shifts in the abundance of gut microbes. Doxycycline is a tetracycline antibiotic widely used for malaria prophylaxis in travelers. We prospectively studied changes in the fecal microbiota of 15 French soldiers after a 4-month mission to Mali with doxycycline malaria prophylaxis, compared to changes in the microbiota of 28 soldiers deployed to Iraq and Lebanon without doxycycline. Stool samples were collected with clinical data before and after missions, and 16S rRNA sequenced on MiSeq targeting the V3-V4 region. Doxycycline exposure resulted in increased alpha-biodiversity and no significant beta-dissimilarities. It led to expansion in *Bacteroides*, with a reduction in *Bifidobacterium* and *Lactobacillus*, as in the group deployed without doxycycline. Doxycycline did not alter the community structure and was specifically associated with a reduction in *Escherichia* and expression of *Rothia*. Differences in the microbiota existed at baseline between military units but not within the studied groups. This group-effect highlighted the risk of a Simpson paradox in microbiome studies.

## 1. Introduction

The gut microbiota has been rediscovered and described by metagenomic methods over the past decade [1]. Its normal constituents are primarily bacteria, the vast majority of which reside in the colon, where densities approach 10^12^ cells/mL [2]. The gut bacterial microbiota interacts with the host immune system and plays key roles, such as the degradation of dietary components, production of vitamins, degradation of xenobiotics, and protection from pathogen invasion. Significant changes to the microbial composition can lead to changes in resource availability and species–species interactions, with clinical and metabolic implications, such as weight gain [3]. Each individual carries their own bacterial community acquired over a lifetime and modulated via exposure to many factors, including diet, lifestyle, physical and psychological stressors, and other environmental sources [2]. More particularly, antibiotics have been associated with consistent alterations to the intestinal microbiota community structure by reducing bacterial diversity and redistributing taxa composition, leading to an intestinal dysbiosis, sometimes opening niches for pathogenic intrusion [4]. To explore the intestinal bacterial ecosystem, fecal samples are commonly analyzed as a surrogate for the entire gut microbiota. Measurement of microbiota diversity integrates the study of richness (number of different species), evenness (dominant and rare species), and disparity (dissimilarity between species) [5]. Diversity is usually studied at two levels: alpha-diversity, which evaluates how diverse one community is (within one group or one ecosystem) and beta-diversity, which represents differences in the abundance (i.e., the presence and count) of species between several communities [6].

Doxycycline is a semisynthetic second-generation tetracycline antibiotic and a schizonticide agent, highly effective for the prevention of malaria in travelers. Doxycycline is especially useful in areas with *Plasmodium falciparum* malaria. Dosing in adults is 100 mg once daily, starting 1–2 days before travel and continued for 4 weeks after leaving the at-risk area [7]. In addition, doxycycline has a broad spectrum of activity against bacteria, so that extended exposure to doxycycline may cause long-lasting effects on gut community composition [8,9]. Moreover, doxycycline long-term use at low-dose (100 mg daily) for malaria prophylaxis has been reported to be protective against digestive infections in travelers [10], suggesting its impact on gut microbiota is worth investigating. In humans, studies by culture have reported minor and rapidly reversible changes [11]. Biomolecular studies on doxycycline at a therapeutic dosage in humans (200 mg daily) found a reduction in diversity of *Bifidobacterium* populations [12] and *Bacteroidetes*, *Firmicutes*, *Lactobacillus*, and total bacteria, these alterations being significantly associated with the duration of treatment [13]. Few studies have described gut changes on doxycycline using 16S RNA metagenomic techniques: in mice, a lower richness and diversity were reported with an increase in the relative abundance of *Bacteroidetes* [14]. Similar results were found in a sample of three humans exposed to tetracyclines at increasing dosage, but the interpretation of results was limited by a very high inter-individual variability [15].

Military personnel are international travelers, providing a rare opportunity among the traveling population to ensure follow-up and to prospectively collect broad clinical and biological data. Their tracking is facilitated by their life and work in a community inside military units. In this work, we studied changes in the bacterial intestinal microbiota of French soldiers after long-term low-dose exposure to doxycycline during a mission to Mali, where malaria prophylaxis is recommended. These variations were compared to changes in the microbiota composition of soldiers concurrently deployed to Iraq and Lebanon without doxycycline malaria prophylaxis. Subjects of the study belonged to four distinct military units grouped in pairs to constitute the two doxycycline groups. Importantly, reasons for the difference observed between two groups may be confounded by an unrecognized other variable. This occurs when, within one group, significant variations between subgroups preexist and lead to erroneous or even reverse conclusions when subgroups are combined, and the outcome of the entire group is compared to another [16,17]. This concept is known as Simpson’s paradox and was more particularly addressed in this work by recruiting participants from different military communities.

## 2. Results

### 2.1. Clinical Characteristics of the Study Population

Overall, 43 soldiers provided fecal samples before (B) and after (A) their mission. Soldiers deployed to Mali originated from two geographically distinct military units (units 1 and 2) and constituted the group exposed to doxycycline (doxy group, n = 15). Soldiers from units 3 and 4 were respectively deployed to Lebanon and Iraq, and constituted the group without doxycycline (nodoxy group, n = 28).

The median mission duration was 129 days, ranging from 117 to 150 days. The population was predominantly athletic young men averaging 30 years old (range (22–34)). Among them, 44% (19/43) were smokers, and 14% (6/43) reported probiotics consumption before the mission. During the mission, 62% (27/43) had rural accommodations most of the time, 76% (33/43) changed their eating habits, 18% (8/43) had sedentary activities, and 48% (21/43) had travel diarrhea, but no participant received antibiotics other than doxycycline during the mission. Stress levels measured by the Spielberger State–Trait Anxiety Inventory (STAI) remained stable before and after the mission [18]. There were no significant clinical differences between the two doxy and nodoxy groups (Table 1 and Table 2).

### 2.2. Changes in Bacterial Community Structure

**Figure 1:**
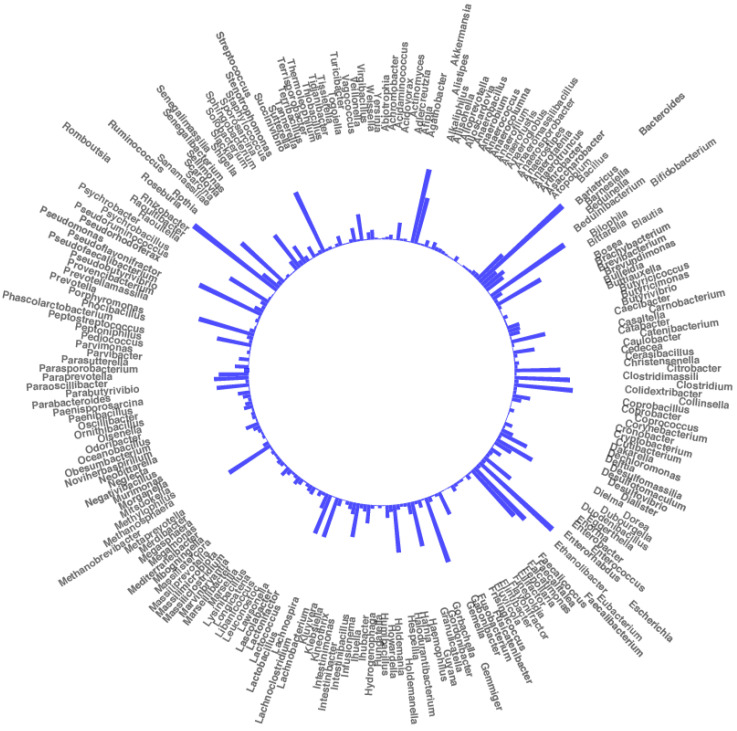
Circular plot representing 233 bacterial genera identified by 16S rRNA metagenomic sequencing across the 86 samples and their proportional abundance (means of total reads).

After merging and filtering, 5,999,643 high-quality sequence reads were generated, sequencing at a mean depth of 69,763 sequences per sample. Rarefaction curves indicated the sequencing depth was adequate (Supplementary Figure S1). Overall, from the 86 stool samples, 1225 assigned operational taxonomic units (OTUs) were obtained, representing 233 different genera (Figure 1). We identified four groups of stool samples according to their study time point and collection group: before doxycycline exposure during mission (doxy_B); before mission without doxycycline exposure (nodoxy_B); after mission and doxycycline exposure (doxy_A); and after mission without doxycycline exposure (nodoxy_A). Absolute abundances per sample and groups are represented in terms of phyla, orders, and TOP 15 most abundant taxa among *Firmicutes*, *Bacteroidetes*, and *Proteobacteria* (Supplementary Figures S2–S6). A remarkable increase in *Bacteroides* among the *Bacteroidetes* and loss of *Pseudomonas* among the *Proteobacteria* was observed after the mission in the two groups with and without doxycycline (Supplementary Figure S7).

**Figure 2:**
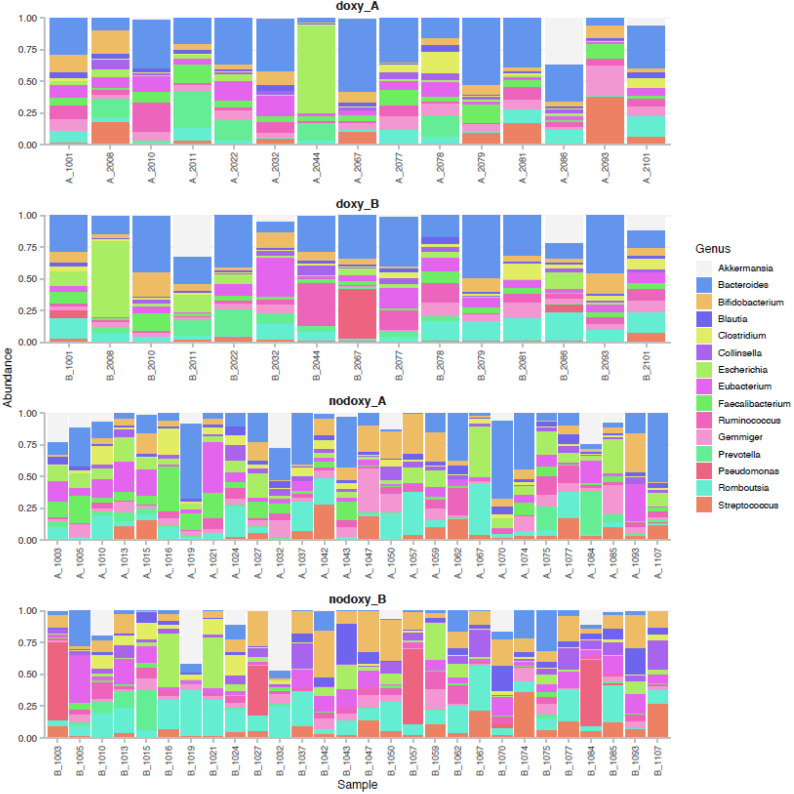
Relative proportion of the TOP 15 most abundant genera per sample and four groups. Numbers are study subject’s ID; (B_) means before mission; (A_) means after mission; (doxy_B) is before mission and doxycycline exposure; (nodoxy_B) is before mission without doxycycline exposure; (doxy_A) is after mission and doxycycline exposure; (nodoxy_A) is after mission without doxycycline exposure.

**Figure 3:**
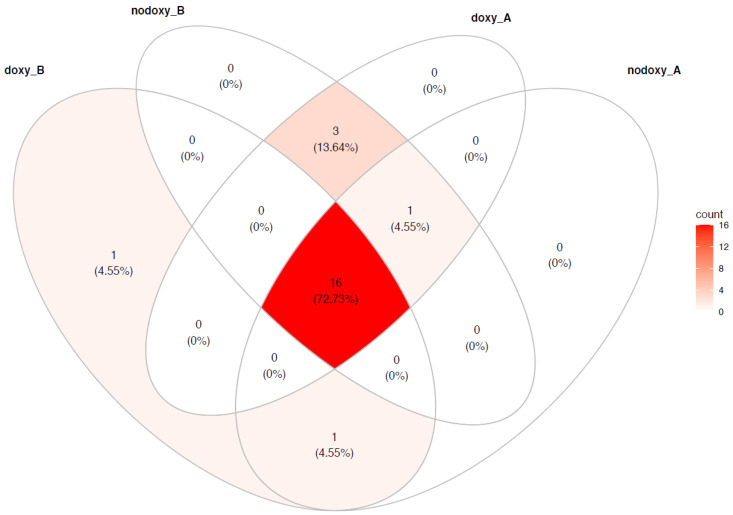
Core microbiomes: each circle contains the number of genera belonging to the core microbiome of the groups being compared. Core microbiome = 22 genera; non-core microbiome = 211 genera. (doxy_B) is before mission and doxycycline exposure; (nodoxy_B) is before mission without doxycycline exposure; (doxy_A) is after mission and doxycycline exposure; (nodoxy_A) is after mission without doxycycline exposure.

Between-subject and within-subject differences in the relative abundance of the TOP 15 genera are depicted per group in Figure 2. The core microbiome of the population was defined by 22/233 (9%) genera, with 73% similarity between the four groups (Figure 3 and Table 3).

### 2.3. Microbial Diversity

**Figure 4:**
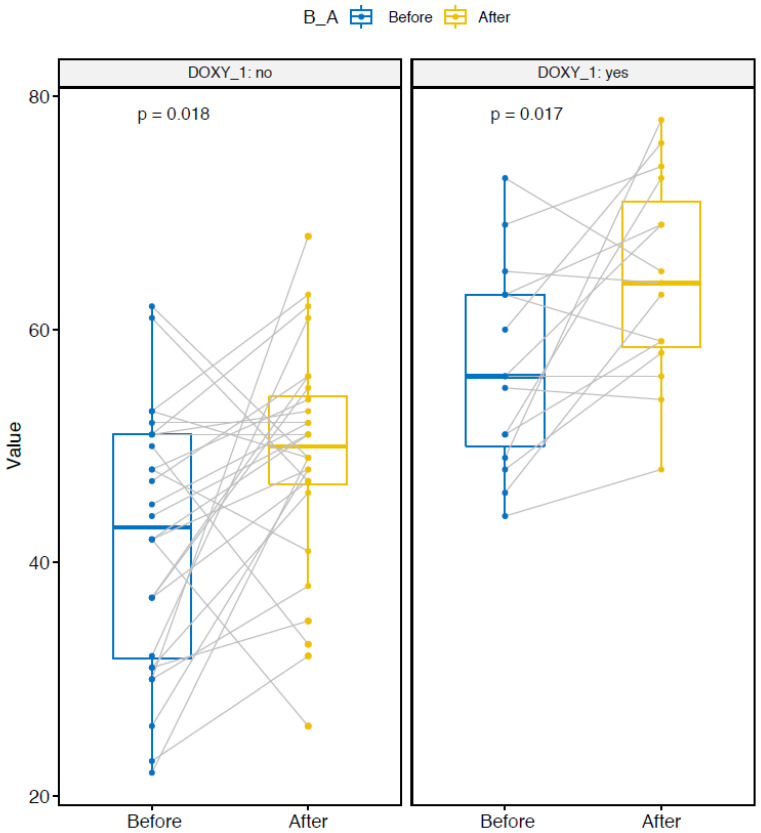
Alpha diversity metrics: Chao1 index variation after deployment in the two groups with doxycycline (DOXY_1 yes) and without doxycycline (DOXY_1 no). Paired samples are connected by grey lines. *p*-values are calculated by ANOVA.

**Figure 5:**
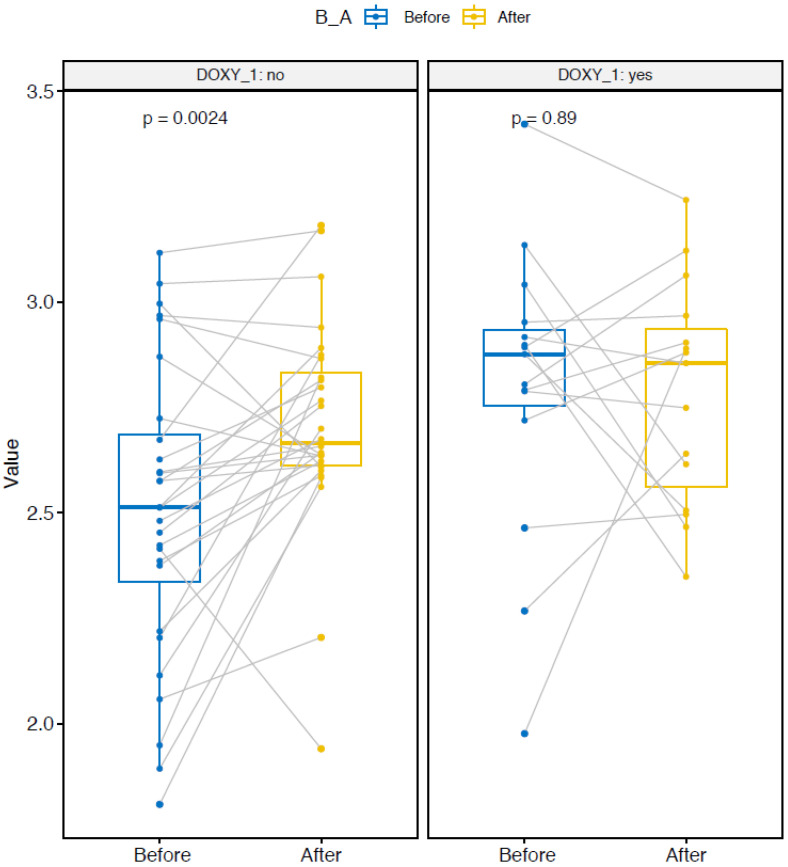
Alpha diversity metrics: Shannon index variation after deployment in the two groups with doxycycline (DOXY_1 yes) and without doxycycline (DOXY_1 no). Paired samples are connected by grey lines. *p*-values are calculated by ANOVA.

**Figure 6:**
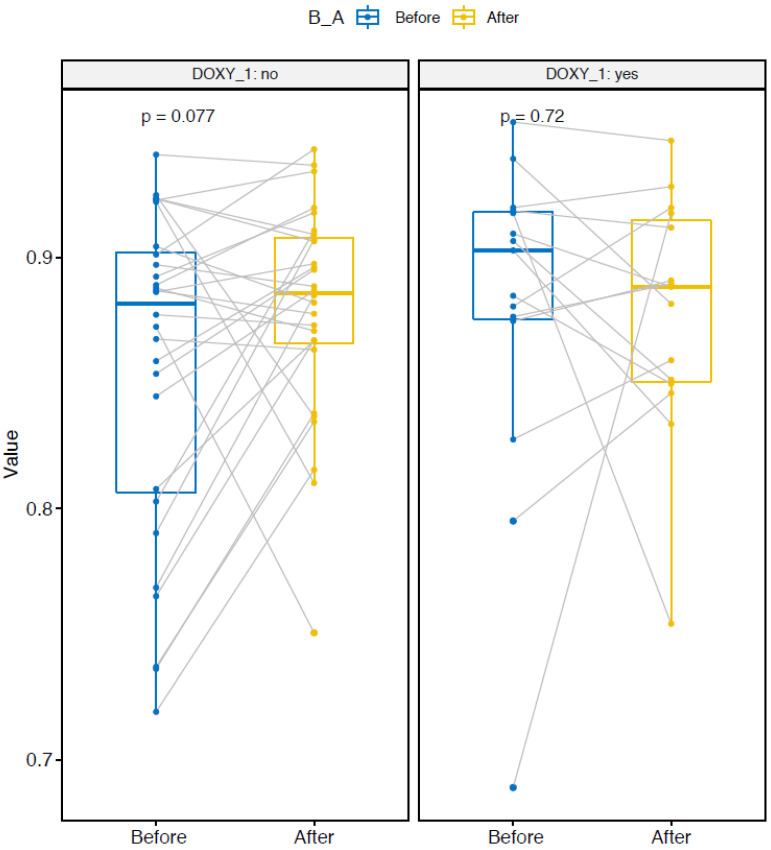
Alpha diversity metrics: Simpson index variation after deployment in the two groups with doxycycline (DOXY_1 yes) and without doxycycline (DOXY_1 no). Paired samples are connected by grey lines. *p*-values are calculated by ANOVA.

The Chao1 nonparametric-richness estimate significantly increased in the two doxy and nodoxy groups (Figure 4) [19]. The Shannon and Simpson diversity indexes are entropy measurements, taking into account both richness and evenness (i.e., equitability between samples) [20,21]. The Shannon index significantly increased only in the group without doxycycline (Figure 5). The Simpson index increased in the nodoxy group, and slightly decreased in the doxy group, but not significantly (Figure 6).

**Figure 7:**
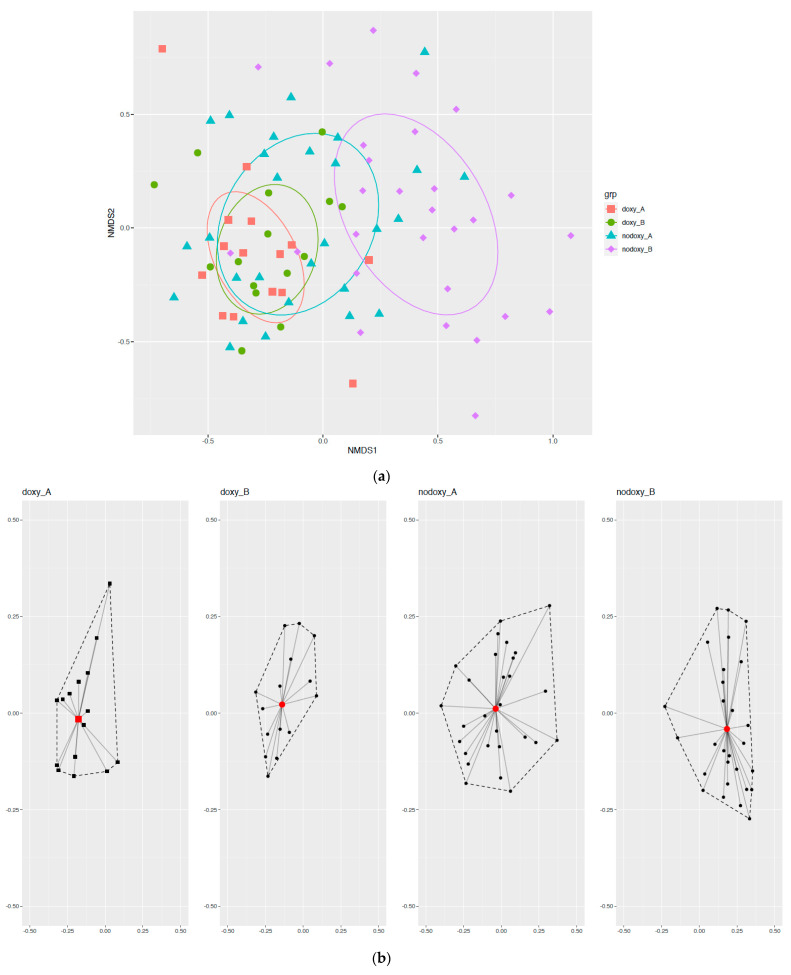

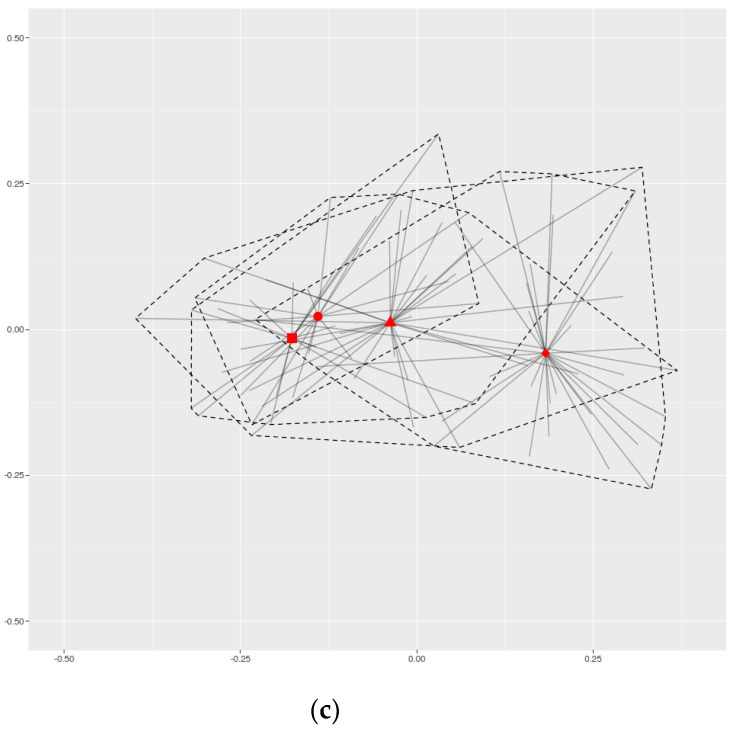
Beta diversity results before and after deployment within and between the two doxycycline groups: (doxy_B) is before doxycycline exposure; (nodoxy_B) is before mission without doxycycline exposure; (doxy_A) is after doxycycline exposure; (nodoxy_A) is after mission without doxycycline exposure: (**a**) nonmetric multidimensional scaling (NMDS) of Bray–Curtis distances; (**b**) Variances intra (within) groups; (**c**) Variances inter (between) groups.

Based on the relative abundance of taxa, beta-diversity analyses by nonmetric multidimensional scaling (NMDS) and variances comparison of Bray–Curtis distances indicated no significant differences in fecal microbiota before and after mission in the doxy group (permutational multivariate analysis of variance (PERMANOVA), *p*-value = 0.39). On the contrary, Bray–Curtis dissimilarities after deployment were significant in the group without doxycycline (nodoxy group) (PERMANOVA, *p*-value = 0.001) (Figure 7).

### 2.4. Group-Effect

**Figure 8:**
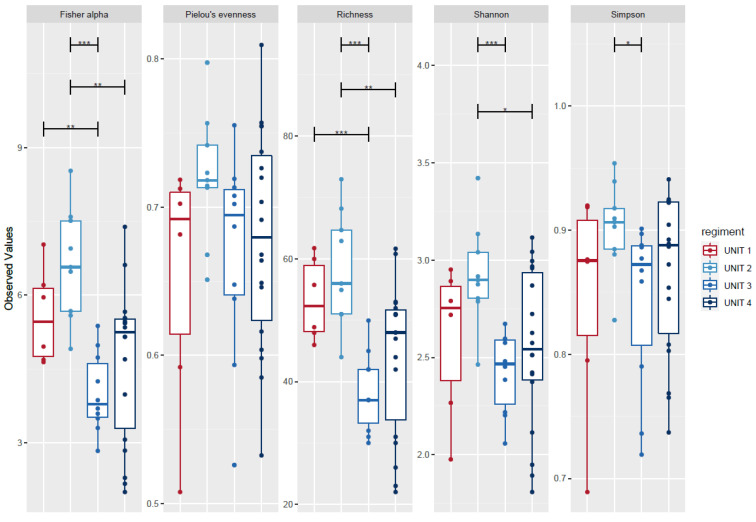
Alpha diversity metrics: Fisher’s alpha, Pielou’s evenness, richness, Shannon and Simpson indexes in samples before deployment with respect to the military units (regiment) compared using ANOVA * *p*-value < 0.05; ** *p*-value = 0.01–0.001; *** *p*-value < 0.001 (subgroup before (B), n = 43). Boxplots represent diversity measures (center line is median, lower, and upper hinges correspond to the first (Q1) and third (Q3) quartiles; the upper whisker is located at the smaller of the maximum alpha diversity measures and Q3 + 1.5 × IQR (Q3 − Q1); the lower whisker is located at the larger of the minimum alpha diversity measures and Q1 − 1.5 × IQR).

Analyses were performed at the subgroup level of military units at baseline to address the risk of Simpson’s paradox. Richness, Fisher’s alpha index, and the Shannon index differed significantly at baseline between some military units, but importantly, no alpha-diversity index differed significantly between the two units of the doxy group (units 1 and 2), or between the two units of the nodoxy group (units 3 and 4) (Figure 8).

**Figure 9:**
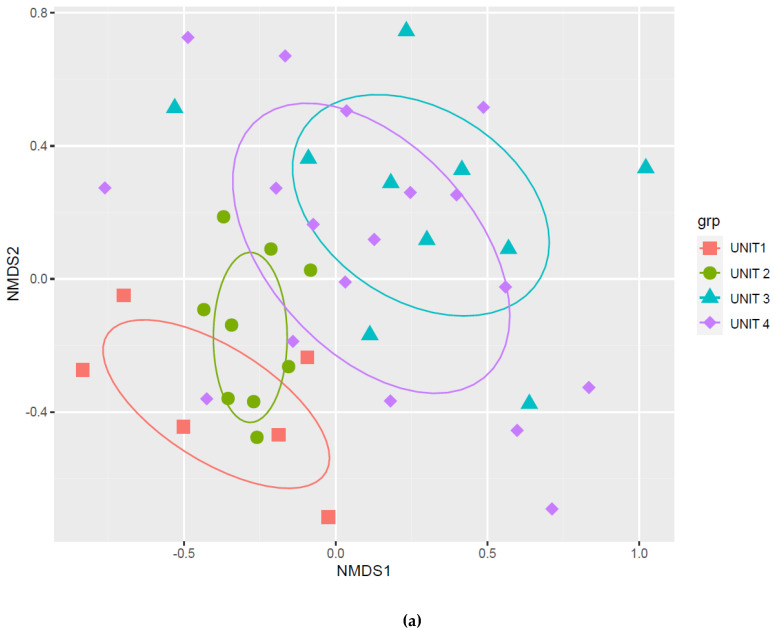

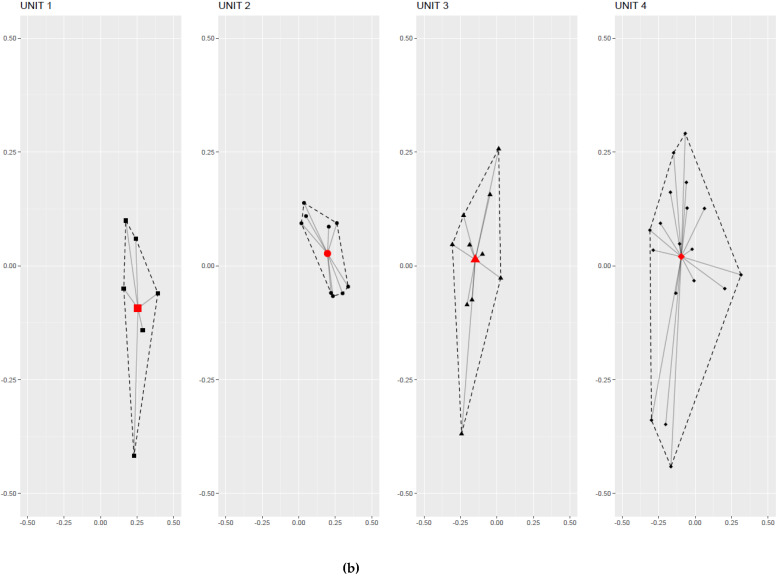

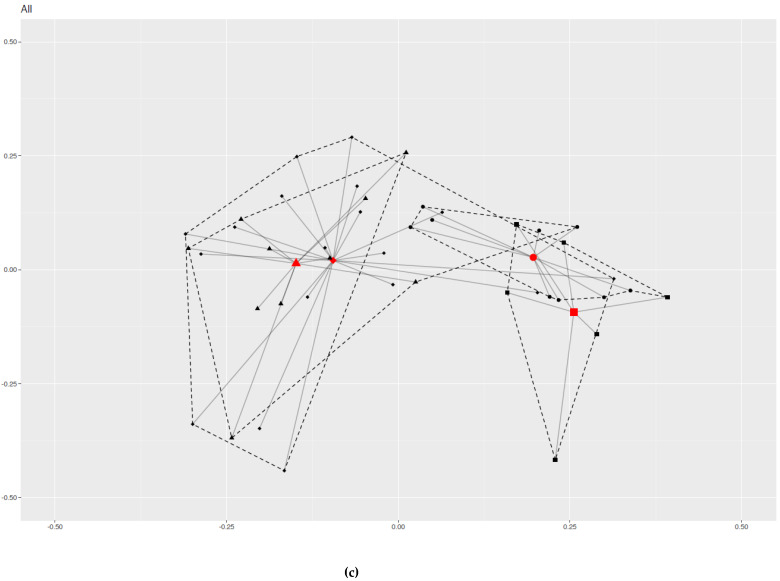
Beta diversity results before deployment with respect to the four military units (subgroup before (B), n = 43): (**a**) nonmetric multidimensional scaling (NMDS) of Bray–Curtis distances; (**b**) Intra-regiment variances; (**c**) Inter-regiment variances.

Likewise, Bray–Curtis dissimilarities were significant at baseline between military units (PERMANOVA, *p*-value = 0.001), but not between units 1 and 2 (*p*-value = 0.05), or between units 3 and 4 (*p*-value = 0.41) (Figure 9). All these results confirmed a major group-effect on military international travelers’ fecal microbiota diversity and the risk of a Simpson paradox occurrence in microbiota studies.

### 2.5. Fecal Microbiota Composition

**Figure 10:**
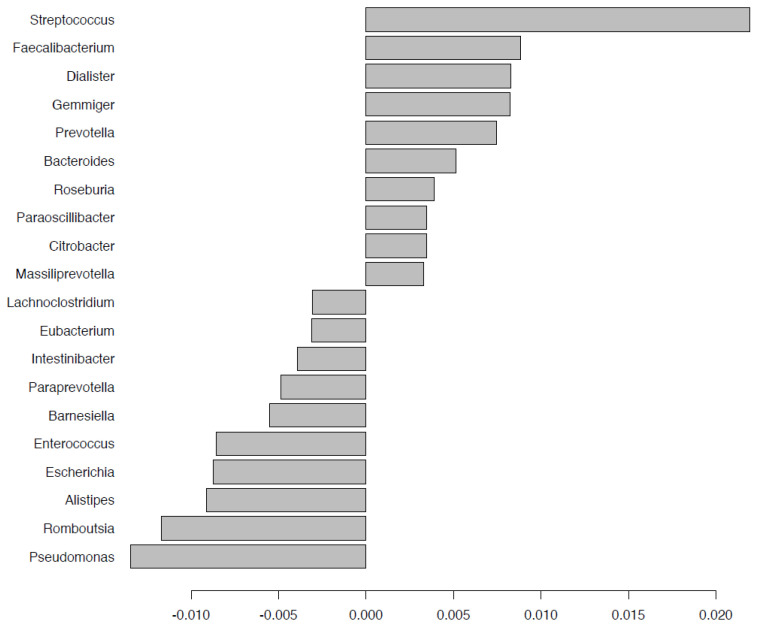
Taxa with the best significant Bray Curtis dissimilarities after (versus before) doxycycline exposure (subgroup doxy, n = 15) using permutational multivariate analysis of variance (PERMANOVA).

**Figure 11:**
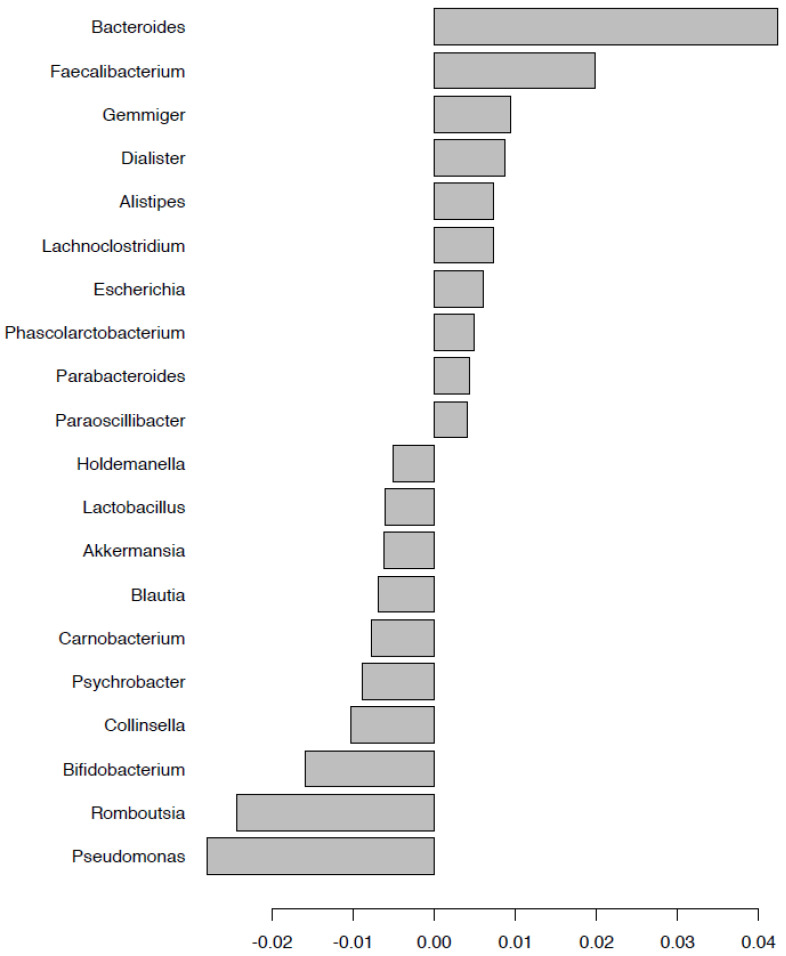
Taxa with the best significant Bray Curtis dissimilarities after deployment (versus before) within the group without doxycycline (subgroup nodoxy, n = 28) using permutational multivariate analysis of variance (PERMANOVA).

**Figure 12:**
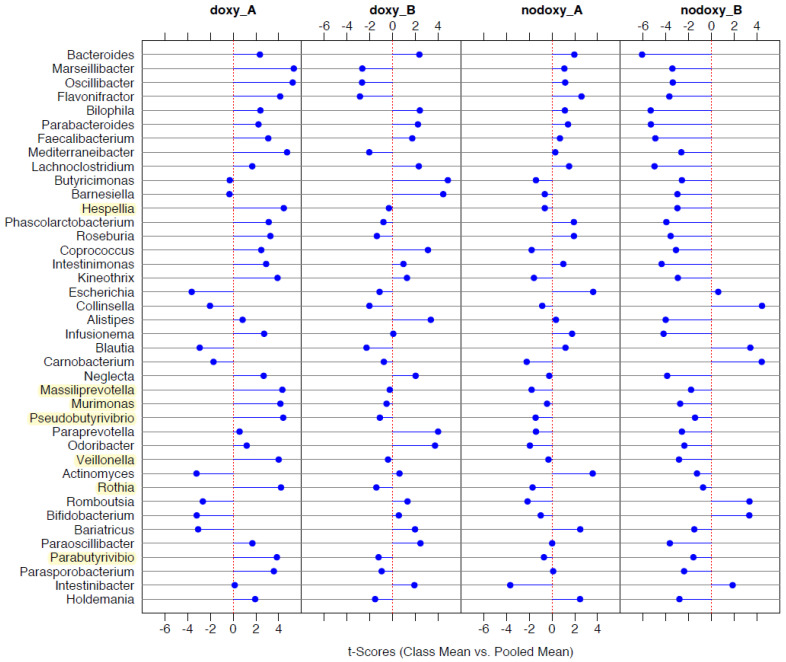
Binary Discriminant Analysis (BINDA) for bacterial taxonomic differentiation between the four groups (n = 43): (doxy_B) is before doxycycline exposure; (nodoxy_B) is before mission without doxycycline exposure; (doxy_A) is after doxycycline exposure; (nodoxy_A) is after mission without doxycycline exposure. Taxa, which differentiated the group doxy_A from each of the other three groups, are highlighted in yellow.

Bacterial taxonomic differentiation after deployment was performed by binary discriminant analysis (BINDA) and PERMANOVA on Bray–Curtis distances in the two groups with and without doxycycline for comparison. After doxycycline exposure during the mission, *Lactobacillus* and *Bifidobacterium* genera were lower; abundance of *Streptococcus*, *Faecalibacterium*, *Bacteroides*, *Roseburia*, and *Massiliprevotella* were significantly increased; *Escherichia*, *Enterococcus*, and *Pseudomonas* were significantly decreased (Figure 10). Among the nodoxy group, *Bacteroides*, *Faecalibacterium*, and *Escherichia* were among the significantly rising taxa after the mission, while *Pseudomonas*, *Lactobacillus*, *Bifidobacterium*, *Romboutsia*, *Collinsella*, and *Akkermensia* were lost (Figure 11). Targeting taxa which differentiated the group after doxycycline exposure (doxy_A) from each of the other three groups in the BINDA (Figure 12), we identified three taxa; *Massiliprevotella*, *Pseudobutyrivibrio*, and *Rothia*, that could specifically correlate with doxycycline exposure. The indicator species analyses confirmed a significant association of *Rothia* genus with the after-doxycycline exposure group (nodoxy group: *Rothia* After = 0.32, Before = 0.70, *p*-value > 0.05; doxy group: *Rothia* After = 0.67, Before = 0.32, *p*-value = 0.008; group after mission: *Rothia* doxy = 0.85, nodoxy = 0.67, *p*-value = 0.001).

### 2.6. Gut Microbiota and Clinical Data Other Than Doxycycline Exposure

Considering the global decrease in *Lactobacillus* and *Bifidobacterium* after the mission in the global population regardless of doxycycline exposure, we searched for a potential link with the interruption of regular probiotic consumption due to deployment. We analyzed abundances of *Lactobacillus* and *Bifidobacterium* before and after deployment, according to the consumption of probiotics contained in fermented dairy products before the mission (probiotics n = 6/43 (14%); no probiotics n = 25/43 (58%); probiotics unknown: n = 12/43 (28%)) (Supplementary Figures S8 and S9). The average read count of *Lactobacillus* was 186 before the mission and 162.3 after the mission in the group consuming probiotics (delta mean after mission = −24), while 350.3 before the mission and 289 after the mission in the group never consuming probiotics (delta mean after mission = −61). The average read count of *Bifidobacterium* was 2574.5 before the mission and 3137.6 after the mission in the group consuming probiotics (delta mean after mission = +563), while 3939.0 before the mission and 2143.6 after the mission in the group never consuming probiotics (delta mean after mission = −1796.0). Globally, soldiers consuming probiotics contained in fermented dairy products at baseline had lower levels of *Lactobacillus* and *Bifidobacterium* before deployment and smaller loss at return in *Lactobacillus* than soldiers never consuming probiotics. They also had an increase in *Bifidobacterium* after the mission, contrary to soldiers who had never consumed probiotics. However, inter- and intra-individual variability in abundances of *Lactobacillus* and *Bifidobacterium* was very high per and between the groups of probiotics consumption.

We compared alpha-diversity between the groups with and without travel diarrhea before and after deployment. We found no significant difference at baseline between the groups (Supplementary Figure S10), whereas at return, soldiers reporting diarrhea had significantly lower Fisher’s alpha, Shannon and Simpson indexes than the others (Supplementary Figure S11). Dissimilarities between the groups were confirmed by Bray–Curtis distances in principal coordinates analysis (PCoA) and PERMANOVA (*p*-value = 0.021) (Supplementary Figure S12). Alpha and beta diversities were analyzed with respect to the following metadata of samples finding no significant differences (n = 43): smoking status (yes or no) (Supplementary Figures S13 and S14); sex (male, female) (Supplementary Figures S15 and S16); and BMI group (normal, overweight or obesity) (Supplementary Figures S17 and S18).

## 3. Discussion

Since the start of the Human Microbiome Project in 2007, many determinants of gut bacterial community composition have been hypothesized, and associations between microbiota profiles and clinical conditions have been extensively reported [22]. However, reasons for the difference between various populations or conditions may not be due only to the factor studied. One major confounding factor can be that within a group, significant variations between subgroups exist, which is the Simpson’s paradox that was illustrated in our work [19]. Indeed, doxycycline exposure depended on the originating military units, but we found that the gut microbiota could significantly differ between military units at baseline. The major strength of this study was to assess not only inter- but also within-group differences by providing each subject with their own control before and after deployment and doxycycline exposure. This design enabled us to find the confounding effect of military units. Moreover, Bray–Curtis dissimilarities after missions were significant in soldiers deployed to Iraq and Lebanon and drew near the doxy group after mission, meaning that individual microbiome stability was affected after a several-month period of deployment in this group. We highlighted here the key role played by the ecosystem to which an individual belongs in the determination and modulation of their intestinal community structure. Both environmental, diet, and behavioral factors interfere and identifying the exact role played by each of them, or another external factors, requires rigorous methods. Studying one effect on the microbiome, all other things being equal (“Ceteris paribus” hypothesis), implies the entire population must share exactly the same ecosystem over time. Multicentric recruitments result in exposure to confounding factors and can even lead to Simpson’s paradox in microbiome explorations [16,17]. These statements mean that study findings strictly apply to the studied populations. Only the reproduction of similar results in Different ecosystems enables their validation and generalization [23].

Like Walters et al., we found great overlaps in the core microbiome of soldiers [24]. However, significant differences in alpha- and beta-diversity between groups existed, suggesting that the core microbiome approach is not extensive enough. We first observed a significant global increase in richness and the Chao1 index after missions in all groups. In soldiers deployed to Iraq and Lebanon without doxycycline, there was also a significant increase in the Shannon index. Beta-dissimilarities at return from baseline were not significant in the subgroup with doxycycline. Thus, although doxycycline malaria prophylaxis is a long-term, low-dose antibiotic regimen, it does not appear to alter the microbiota structure. The doxycycline impact on the gut microbiota is probably dose-dependent [11]. Indeed, no effect on the composition of the fecal microbiota was found in humans at prolonged suboptimal dosage (20 mg per day for 9 months) [25], and only a therapeutic dosage of tetracycline induced changes in the fecal microbiome of broiler chickens [26].

Deployment on doxycycline malaria prophylaxis was significantly and specifically associated with the presence of *Rothia* (*R.*) in our population. At the species level, this corresponded to the presence of *R. mucilaginosa*, sometimes associated with *R. dentocariosa*. *Rothia* is a Gram-positive, aerobic or facultatively anaerobic, rod-shaped, and non-motile bacterial genus from the family of *Micrococcaceae* and the phylum of *Actinobacteria*. *Rothia* species were first misidentified as Gram-positive bacilli (*Actinomyces* spp., *Nocardia* spp.), or Gram-positive cocci (*Micrococcus*, *Staphylococcus*), until Georg and Brown proposed the new genus *Rothia* in 1967 in recognition of Genevieve Roth’s research [27,28]. It is a commensal of the human oropharynx, upper respiratory tract, and duodenum [29,30]. There are three human species: *R. aeria*, *R. dentocariosa*, and *R. mucilaginosa*, respectively isolated in 2003, 1949, and 1900 [31,32,33]. All three can cause disease in humans, mainly in the oral cavity, and also invasive infections, such as endocarditis [33]. Recently, *Rothia* spp has been linked with the emergence and persistence of gastric atrophy and intestinal metaplasia after *Helicobacter pylori* eradication [34]. Moreover, *R. mucilaginosa*, previously known as *Stomatococcus mucilaginosus*, produces enterobactin, a metal-chelating siderophore of *Escherichia coli* [35]. We suggest *Rothia* could promote doxycycline-related esophagogastric mucosal injury [36]. This observation warrants further studies.

Doxycycline and deployment were associated with increased amounts of genera, such as *Hespellia*, *Murimonas*, *Roseburia*, *Pseudobutyrivibrio*, and *Parabutyrivibrio*, which are members of the family *Lachnospiraceae*, within the order *Clostridiales*, among the *Firmicutes.* They include core species responsible for butyrate production, as well as flagellin-bearing bacteria. Changes in the occurrence of members of the *Lachnospiraceae* have been associated with inflammation of the gut and colitis, some species being beneficial, e.g., *Roseburia* spp, and others being potentially harmful [37,38,39]. Importantly, the hierarchical levels at which taxonomic units become biologically meaningful are not well defined, and the health effects of within-taxa variations remain unclear.

In the whole population, deployment was associated with an increase in *Bacteroides*, *Faecalibacterium*, and *Prevotella*, and a decrease in *Bifidobacterium* and *Lactobacillus.* It is highly probable that diet elicited these changes [40,41]. We could not address this point because we did not monitor food habits during the mission, while 77% of soldiers reported dietary changes during deployment. Consumption of probiotics contained in fermented dairy products at baseline did not affect *Lactobacillus* and *Bifidobacterium* abundances before deployment and was not associated with a greater loss of *Lactobacillus* and *Bifidobacterium* after the interruption of probiotic consumption during the mission period, suggesting the poor participation of oral supplementation in the abundance of these genera.

Interestingly, Karl et al. reported a decrease in lactic acid bacteria among *Firmicutes* in individuals consuming military food rations [42]. The genus *Bacteroides* is one of the most dominant bacterial groups in the human colon and includes anaerobic, Gram-negative, non-spore-forming, and rod-shaped bacteria involved in many important metabolic activities in the human colon such as hydrolysis of polysaccharides and carbohydrate fermentation. In studies linking dietary patterns with the gut microbiota, *Bacteroides* enterotype was highly associated with animal protein and saturated fats, while the *Prevotella* enterotype was associated with a high level of carbohydrates [43,44]. Thus, a low-fiber diet, likely with the consumption of military food rations as soldiers were in the field most of the time, presumably explains the microbiota profile observed after deployment.

On another note, Karl et al. reported a reduction in *Lactobacillus* and *Bifidobacterium* abundances in military-relevant rodent stress models [45] and a reduction in *Bacteroides* in soldiers under multiple-stressor military training environments [46]. Measuring physical and psychological indexes before and after deployment, we found no significant variations. However, the results revealed a high level of anxiety among our military population at baseline, especially in the group assigned to the mission in Mali, where conditions are currently harsher than in other theaters of operation [47]. Apparently, the combat environment and psychological and physical stressors participate in the ecosystem that modulates soldiers’ gut microbiota, but their impact still needs to be characterized among different populations.

Lastly, diversity dissimilarities did not depend on sex, BMI, and smoking status. Subgroup analyses in soldiers reporting travel diarrhea, found significantly lower Shannon and Simpson indexes after diarrhea, suggesting post-diarrhea dysbiosis. We observed diminished levels of *Escherichia* in the doxycycline group, which corroborates previous results obtained by culture of human fecal samples during 100 mg daily doxycycline regimens [8]. In this small population sample, the prevalence of travel diarrhea was comparable between the two groups with and without doxycycline. However, in larger populations, doxycycline malaria prophylaxis protected against travel diarrhea [10]. Our results support the hypothesis that doxycycline may have restored antimicrobial properties against enteropathogenic bacterial strains and protect against travel diarrhea by direct inhibition of *Escherichia*. We will conduct a specific analysis in this cohort to describe gut microbiota profiles before and after diarrhea in comparison with individuals not reporting diarrhea. We intend to identify taxa predictors of diarrhea occurrence or non-occurrence and to characterize post-travel diarrhea microbiota.

## 4. Materials and Methods

### 4.1. Study Design and Population

We conducted a prospective cohort analysis among four military units in France in 2016–2018. All French military personnel assigned to a mission were eligible. Volunteers were enrolled in the month prior to deployment. Two clinical visits were planned: at the time of inclusion and within one month after return. Exclusion criteria were pregnancy, antibiotics administration in the last 3 months, recent diarrhea (<4 weeks), cancer, inflammatory bowel diseases, and contraindication to doxycycline.

### 4.2. Clinical Data Collection

At the two visits, participants completed questionnaires recording socio-demographic information, smoking status, physical activity (sport hours per week), medical events, and medications. Participants filled in the STAI, and a cut-off point of 40 was considered as the detection of significant symptoms of stress and anxiety [18]. Weight (kg), height (cm), and waist circumference (cm) were measured by study investigators at each visit. The BMI was calculated by dividing a person’s weight in kilograms by the square of their height in meters (kg/m^2^). People with a BMI greater than or equal to 25 were overweight, and people with a BMI over 30 were obese. Acute travel diarrhea was defined as three or more loose stools in 24 h or two loose stools in 24 h associated with other gastrointestinal symptoms during the mission. Upon return, participants were questioned on their accommodations for most of the mission time (i.e., urban if in hard-wall constructions versus rural if in rudimentary housing or tents), the occurrence and duration of diarrhea, any administration of an antibiotic other than doxycycline, and doxycycline observance when applicable. The population was split into two groups based on the military guidelines for their region of travel. Military deployed in Mali received doxycycline malaria prophylaxis at 100 mg daily, continued for one month after return (“doxy” group), and those in Iraq or Lebanon had no chemoprophylaxis (“nodoxy” group).

### 4.3. Sample Collection and Storage

Fresh stools were collected before the mission (group B) and after the mission (group A) and transported at +4 °C to the IHU-Méditerranée Infection laboratory for aliquoting in sterile screw cap 2 mL microtubes, with storage at −80 °C until analysis. We identified 4 groups of stool samples: collected before mission and doxycycline exposure (doxy_B); before mission without doxycycline exposure (nodoxy_B); after mission and doxycycline exposure (doxy_A), and after mission without doxycycline exposure (nodoxy_A).

### 4.4. DNA Extraction

We used our lab’s protocol based on glycoprotein lysis to get maximal diversity [48]. We first added 500 μL of PBS to 0.25 g of stool and homogenized it with the high-speed digitally controlled benchtop homogenizer FastPrep^®^ (Biomedicals, Santa Ana, California, USA). Then, 200 μL of this mixture was centrifuged at 17,000 rpm for 10 min. After supernatant removal, we resuspended it in 20 μL of 10X Glycoprotein Denaturing Buffer (New England Biolabs) and denatured the glycoproteins by heating reaction at 100 °C for 10 min. We then added 160 μL of H_2_O and 40 μL of 10X G5 reaction buffer, followed by 5 µL of EndoHf (New England Biolabs, Ipswich, Massachusetts, USA), 5 μL of cellulase (SIGMA, Saint-Quentin-Fallavier, France), and 5 μL of PNGase F (SIGMA, Saint-Quentin-Fallavier, France). The mixture was incubated overnight at 37 °C before DNA extraction using the EZ1 Advanced XL automate and the QIAamp^®^ DNA Stool Mini Kit (Qiagen, Courtaboeuf, France), according to the manufacturer’s instructions [49].

### 4.5. Metagenomic Sequencing

Fecal samples were amplified, barcoded, pooled, and 16S rRNA sequenced on MiSeq technology (Illumina, Inc., San Diego, CA, USA) with a paired-end strategy, constructed according to the Nextera XT library kit (Illumina, San Diego, USA). For sequencing, DNA was amplified for the 16S “V3-V4” regions by PCR, using the Phusion Taq (ThermoFisher Scientific Inc, Waltham, MA, USA) and the surrounding conserved region V3_V4 primers with overhang adapters (FwOvAd_341F TCGTCGGCAGCGTCAGATGTGTATAAGAGACAGCCTACGGGNGGCWGCAG; RevOvAd_785R GTCTCGTGGGCTCGGAGATGTGTATAAGAGACAGGACTACHVGGGTATCTAATCC). After purification on AMPure beads (Beckman Coulter Inc., Fullerton, CA, USA), the concentration was measured using high sensitivity Qubit technology (Beckman Coulter Inc., Fullerton, CA, USA) and dilution to 0.2 ng/μL was performed. Using a subsequent limited cycle PCR on 1 ng of cleaned PCR product, Illumina sequencing adapters and dual-index barcodes were added to the amplicon. After purification on AMPure beads (Beckman Coulter Inc., Fullerton, CA, USA), the library was then normalized by beads according to the Nextera XT protocol (Illumina Inc., San Diego, CA, USA). Each sample was pooled with other multiplexed samples into a single library for sequencing on MiSeq (Illumina Inc., San Diego, CA, USA). Automated cluster generation and paired-end sequencing with dual index reads were performed in a single 39-h run in a 2 × 251 bp. Total information of 4.4 Gb was obtained from a 1130 K/mm^2^ cluster density, with a cluster passing quality control filters of 37.1% (22,849,000 clusters). Within this run, the index representation was determined with an average of 0.8%. The raw data were configured in fastq files for R1 and R2 reads.

### 4.6. Metagenomic Bioinformatics

#### 4.6.1. Reads Analysis

The corresponding paired-end sequences from the Illumina Miseq raw fastq files were prepared and analyzed for read quality using VSEARCH [50]. Primers were removed and reads of poor quality were filtered by Cutadapt. Paired-end sequences were merged into longer sequences using PANDAseq and filtered for quality with VSEARCH to keep sequences with a length shorter than 500 nts and longer than 200 nts.

#### 4.6.2. Clusterization and Taxonomic Assignment

We used quantitative insights into microbial ecology (QIIME, Knight and Caporaso labs, Northern Arizona University, Flagstaff, AZ, USA.) software [51]. Chimeric sequences were removed using the ChimeraSlayer from QIIME. These filtered sequences were merged and clustered into OTUs with method at 97% similarity, without considering the singletons (add_qiime_label, pick_otus, and pick_rep_set_script). OTUs were then searched against the 16S rRNA databases Silva and culturomics (local database) with the basic local alignment search tool for nucleotides (BLASTN) [52,53]. QIIME uses the Ribosomal Database Project (RDP) classifier to assign taxonomic data to each representative sequence from stage [54]. Reads exhibiting a sequence similarity over 97% and a 100% sequence coverage using the BLASTN algorithm were identified at the species level with the best hit; matchings between 95 and 97% identity were identified at the genus level; between 90 and 95% at the family level and less than 90% at the kingdom level. Lack of a hit led to unassigned OTUs.

#### 4.6.3. Filters and Corrections

To correct inflation in OTUs, we used the AmpliconNoise program integrated into QIIME, which removed OTUs never reaching more than 20 reads per sample. To reduce the number of false-positive hits and increase the accuracy of abundance estimates, we used a normalization method based on a Negative Binomial model available in the RNA-Seq focused R package DESeq. We removed unassigned OTUs and OTUs unassigned at a genus level. Considering metagenomic biases, genus was the lowest taxonomic level used for analyses in this study. Taxa were reviewed and corrected according to the NCBI taxonomy database as the source for nomenclature and classification [55].

### 4.7. Metagenomic Bioanalysis

We used R version 4.0.2 software (Ref: R Core Team. *R: A Language and Environment for Statistical Computing*; R Foundation for Statistical Computing: Vienna, Austria, 2020. URL https://www.R-project.org/, accessed on 23 June 2020) [56]. We used the missMDA R-package to handle missing clinical values with multivariate data analysis [57]; the DESeq2 R-package for normalization and differential analysis of count [58]; the iNEXT (iNterpolation/EXTrapolation), which provides simple functions to compute and plot the seamless rarefaction and extrapolation sampling [59], and the phyloseq, microbiome, vegan, ComplexHeatmap R-package for microbiome analyses [60].

Considering the species delimitation problems, together with sampling and metagenomic bias, we worked at the genus level. This approach is cost-effective and improves the reliability of results [61,62]. Moreover, it integrates distances and discrepancies between OTUs by merging them into similar or distinct genera, implementing a phylogenetic dimension in diversity measures. The number of reads assigned to a given genus was calculated per group. Diversity within a community (alpha-diversity) was assessed using the richness, and Fisher’s alpha, Pielou’s evenness, Chao 1, and Shannon and Simpson indexes [19,20,21,63,64]. Testing for significant differences in alpha-diversity indexes between groups and time points was performed by two-way ANOVA. Hierarchical clustering was conducted using the similarity index of Bray–Curtis based on read abundances [65]. Diversity between communities (beta-diversity) was assessed by Bray–Curtis distances visualized by PCoA or converted to ranks for NMDS. PERMANOVA was used to test variables that best represented patterns in the data and identify taxa with significant Bray–Curtis dissimilarities between different conditions [66].

The core microbiome is the number and the identity of taxa that are shared among different individuals of the same group, and is hypothesized to play a key role in this ecosystem [22,67]. The core microbiota of the four groups were compared by Venn diagram, using the function “core_members” of the microbiome R-package [68].

Quantitative data from reads were binarized according to a threshold that best separated the studied groups, and BINDA was used for descriptive bacterial taxonomic differentiation between the groups [69]. Using the indicspecies R-package (ver. 1.7.8), taxa indicators of study groups in terms of frequency, abundance, and exclusiveness were identified and tested for significance with Pearson’s Phi coefficient of association correlation [70].

### 4.8. Statistical Analysis

Clinical continuous variables were reported as median and interquartile range (IQR). Comparisons were conducted using Fisher’s exact test for proportions and the ANOVA or non-parametric Kruskal–Wallis test (when the conditions of use for ANOVA test were not met) to compare means. Conditions with *p*-values ≤ 0.05 were considered significant for all analyses.

### 4.9. Ethics

The study protocol was supported by the French Armed Forces Health Services and approved by the ethics committee (CPP), and by the Agence Nationale de Sécurité du Medicament in France (IDRCB: No. 2015–A01961–48, Ref Promoteur 2015RC0). Written informed consent was obtained with a signature from each volunteer before inclusion.

## Figures and Tables

**Table 1:** Clinical characteristics of the study population (n = 43): comparison between the doxycycline (doxy) and no doxycycline (nodoxy) groups. Values are effective (% in column) unless otherwise indicated. Baseline means before deployment. Fisher’s exact test was used to compare proportions and a non-parametric test for median comparison. In bold are variables with *p*-value < 0.05. IQR means interquartile range, kg is kilograms, STAI is Spielberger State–Trait Anxiety Inventory, BMI is body mass index.

Variables	NODOXY n = 28	DOXY n = 15	*p*-Value
Military units			<0.001
Unit 1	-	6 (40)	
Unit 2	-	9 (60)	
Unit 3	10 (36)	-	
Unit 4	18 (64)	-	
Mission locations			<0.001
Mali	-	28 (100)	
Iraq	18 (64)	-	
Lebanon	10 (36	-	
Age, years, median (IQR)	29.5 (22.7–34.7)	30 (27–34)	0.6
Sex, F/M	2/26	0/15	0.56
BMI class			1
Normal < 25	16 (57)	9 (60)	
Overweight ≥ 25	11 (39)	5 (33)	
Obesity > 30	1 (4)	1 (7)	
Military rank, manager	13 (46)	8 (53)	0.75
Marital status, married or attached	15 (54)	9 (60)	0.75
Active smoker	12 (43)	7 (47)	0.75
Probiotics intake			1
Yes	4 (14)	2 (13)	
No	16 (57)	9 (60)	
Unknown	8 (29)	4 (27	
Baseline sport, hours/week, median (IQR)	6 (3–8.25)	6 (5–9)	0.60
Sport in mission, hours/week, median (IQR)	5 (2–8.25)	7 (4–10)	0.15
Accommodation during mission, urban/rural	11/17	5/10	0.75
Diarrhea during mission	16 (57)	5 (33)	0.20
Sedentary activity during mission	7 (25)	1 (7)	0.23
Change in food habits during mission	23 (82)	10 (67)	0.28
Percentage of weight variation after mission, kg, median (IQR)	0 (−3.25–+1.25)	0 (−1–+1)	0.49
BMI variation after mission, kg/m^2^, median (IQR)	0 (−1.05–+0.38)	0 (−0.3–+0.3)	0.48
Baseline STAI score (0–80), median (IQR)	44.5 (42.5–46.25)	46 (43–49)	0.17
Return STAI score (0–80), median (IQR)	45 (41–47)	44 (43–48.5)	0.19

**Table 2:** Clinical characteristics of the study population (n = 43): comparison before and after the mission in the doxycycline group (doxy) and in the group without doxycycline (nodoxy). Values are effective (% in column) unless otherwise indicated. Baseline means before deployment. Fisher’s exact test was used to compare proportions and a non-parametric test for median comparison. IQR means interquartile range, kg is kilograms, STAI is Spielberger State–Trait Anxiety Inventory, BMI is body mass index.

Variables	NODOXY			DOXY		
	Before Mission n = 28	After Mission n = 28	*p*-Value	Before Mission n = 15	After Mission n = 15	*p*-Value
Weight, kg, median (IQR)	72.5 (68–81)	72 (67.2–83)	0.79	76 (70–81.5)	74 (70.5–80)	0.95
BMI, kg/m^2^, median (IQR)	24.4 (22.9–26.8)	24.1 (22.9–26.2)	0.73	24.5 (23.5–25.6)	24.5 (22.8–26.1)	1
BMI class			0.71			1
Normal < 25	16 (57)	16 (57)		9 (60)	9 (60)	
Overweight ≥ 25	11 (39)	11 (39)		5 (33)	5 (33)	
Obesity > 30	1 (4)	1 (4)		1 (7)	1 (7)	
Sport, hours/week, median (IQR)	6 (3–8.25)	5 (2–8.25)	0.35	6 (5–9)	7 (4–10)	0.79
STAI score (0–80), median (IQR)	44.5 (42.5–46.25)	45 (41–47)	0.98	46 (43–49)	44 (43–48.5)	0.60

**Table 3:** Core microbiome composition and distribution per group (*X* means present).

	Doxy		Nodoxy	
	Before	After	Before	After
*Alistipes*	-	*X*	*X*	-
*Bacteroides*	*X*	*X*	*X*	*X*
*Bifidobacterium*	*X*	*X*	*X*	*X*
*Blautia*	*X*	*X*	*X*	*X*
*Citrobacter*	*X*	-	-	-
*Clostridium*	*X*	*X*	*X*	*X*
*Collinsella*	*X*	*X*	*X*	*X*
*Dorea*	*X*	*X*	*X*	*X*
*Escherichia*	*X*	*X*	*X*	*X*
*Eubacterium*	*X*	*X*	*X*	*X*
*Faecalibacterium*	*X*	*X*	*X*	*X*
*Fusicatenibacter*	*X*	*X*	*X*	*X*
*Gemmiger*	*X*	*X*	*X*	*X*
*Guyana*	*X*	*X*	*X*	*X*
*Intestinibacter*	*X*	-	-	*X*
*Lachnoclostridium*	-	*X*	*X*	-
*Parabacteroides*	-	*X*	*X*	*X*
*Romboutsia*	*X*	*X*	*X*	*X*
*Roseburia*	-	*X*	*X*	-
*Ruminococcus*	*X*	*X*	*X*	*X*
*Senegalimassilia*	*X*	*X*	*X*	*X*
*Streptococcus*	*X*	*X*	*X*	*X*

## Data Availability

The data presented in this study are available on request from the corresponding author. The data are not publicly available due to institutional restriction and confidentiality.

## Supplementary Materials

The following are available online at https://www.mdpi.com/article/10.3390/pathogens10081063/s1: Supplementary Figure S1: Rarefaction curves for samples sequencing depth assessment, using the R package iNEXT (iNterpolation/EXTrapolation), which provides simple functions to compute and plot the seamless rarefaction and extrapolation sampling curves for the three most widely used members of the Hill number family. Supplementary Figure S2: Abundances of phyla per sample and doxycycline groups. Numbers are study subject’s ID; (B_) means before mission; (A_) means after mission; yes is doxycycline exposure during the mission; no is without doxycycline exposure during the mission. Supplementary Figure S3: Abundances of orders per sample and doxycycline groups. Numbers are study subject’s ID; (B_) means before mission; (A_) means after mission; yes is doxycycline exposure during the mission; no is without doxycycline exposure during the mission. Supplementary Figure S4: Read count of TOP 15 most abundant genera among Firmicutes per sample and doxycycline groups. Numbers are study subject’s ID; (B_) means before mission; (A_) means after mission; yes is doxycycline exposure during the mission; no is without doxycycline exposure during the mission. Supplementary Figure S5: Read count of TOP 15 most abundant genera among Bacteroidetes per sample and doxycycline groups. Numbers are study subject’s ID; (B_) means before mission; (A_) means after mission; yes is doxycycline exposure during the mission; no is without doxycycline exposure during the mission. Supplementary Figure S6: Read count of TOP 15 most abundant genera among Proteobacteria per sample and doxycycline groups. Numbers are study subject’s ID; (B_) means before mission; (A_) means after mission; yes is doxycycline exposure during the mission; no is without doxycycline exposure during the mission. Supplementary Figure S7: Heatmap: absolute abundances of the 233 taxa per each of the 86 fecal samples. Numbers are study subject’s ID; (B_) means before mission; (A_) means after mission; (doxy_B) is before mission and doxycycline exposure; (nodoxy_B) is before mission without doxycycline exposure; (doxy_A) is after mission and doxycycline exposure; (nodoxy_A) is after mission without doxycycline exposure. Supplementary Figure S8: Abundance of *Lactobacillus* in the microbiota of soldiers before and after deployment according to their consumption of probiotics before the mission (probiotics yes, n = 6; probiotics no, n = 25; probiotics unknown, n = 13). Supplementary Figure S9: Abundance of *Bifidobacterium* in the microbiota of soldiers before and after deployment according to their consumption of probiotics before the mission (probiotics yes, n = 6; probiotics no, n = 25; probiotics unknown, n = 13). Supplementary Figure S10: Alpha diversity metrics: Fisher’s alpha, Pielou’s evenness, richness, Shannon and Simpson indexes before deployment with respect to the two groups travel diarrhea yes and no (subgroup after mission (A), n = 43). Boxplots represent diversity measures (center line is median, lower, and upper hinges correspond to the first (Q1) and third (Q3) quartiles; the upper whisker is located at the smaller of the maximum alpha diversity measures and Q3 + 1.5 × IQR (Q3 − Q1); the lower whisker is located at the larger of the minimum alpha diversity measures and Q1 − 1.5 × IQR). Comparison is done using ANOVA * *p*-value < 0.05; ** *p*-value = 0.01–0.001; *** *p*-value < 0.001. Supplementary Figure S11: Alpha diversity metrics: Fisher’s alpha, Pielou’s evenness, richness, Shannon and Simpson indexes after deployment with respect to the two groups travel diarrhea yes and no (subgroup after mission (A), n = 43). Boxplots represent diversity measures (center line is median, lower, and upper hinges correspond to the first (Q1) and third (Q3) quartiles; the upper whisker is located at the smaller of the maximum alpha diversity measures and Q3 + 1.5 × IQR (Q3 − Q1); the lower whisker is located at the larger of the minimum alpha diversity measures and Q1 − 1.5 × IQR). Comparison is done using ANOVA * *p*-value < 0.05; ** *p*-value = 0.01–0.001; *** *p*-value < 0.001. Supplementary Figure S12: Beta diversity results: principal coordinates analysis (PCoA) of Bray–Curtis distances with respect to groups before (B) and after (A) mission and with or without diarrhea during the mission (diarrhea_miss). Each point represents one sample. Each color indicates sample metadata diarrhea yes or no. Supplementary Figure S13: Alpha diversity metrics: Fisher’s alpha, Pielou’s evenness, richness, Shannon and Simpson indexes with respect to the two groups of smoking status (smoker yes or no). Boxplots represent diversity measures (center line is median, lower, and upper hinges correspond to the first (Q1) and third (Q3) quartiles; the upper whisker is located at the smaller of the maximum alpha diversity measures and Q3 + 1.5 × IQR (Q3 − Q1); the lower whisker is located at the larger of the minimum alpha diversity measures and Q1 − 1.5 × IQR). Comparison is done using ANOVA * *p*-value < 0.05; ** *p*-value = 0.01–0.001; *** *p*-value < 0.001. Supplementary Figure S14: Beta diversity results: principal coordinates analysis (PCoA) of Bray–Curtis distances with respect to two groups of smoking status (smoker yes or no). Each point represents one sample. Each color indicates sample metadata of smoking status. Supplementary Figure S15: Alpha diversity metrics: Fisher’s alpha, Pielou’s evenness, richness, Shannon and Simpson indexes with respect to the two groups of sex (F Female and M male). Boxplots represent diversity measures (center line is median, lower, and upper hinges correspond to the first (Q1) and third (Q3) quartiles; the upper whisker is located at the smaller of the maximum alpha diversity measures and Q3 + 1.5 × IQR (Q3 − Q1); the lower whisker is located at the larger of the minimum alpha diversity measures and Q1 − 1.5 × IQR). Comparison is done using ANOVA * *p*-value < 0.05; ** *p*-value = 0.01–0.001; *** *p*-value < 0.001. Supplementary Figure S16: Beta diversity results: principal coordinates analysis (PCoA) of Bray–Curtis distances with respect to the two groups of sex (F Female and M male). Each point represents one sample. Each color indicates sample metadata of sex. Supplementary Figure S17: Alpha diversity metrics: Fisher’s alpha, Pielou’s evenness, richness, Shannon and Simpson indexes with respect to the three groups of body mass index class (BMIclass): normal, over (i.e., overweight), and obese. Boxplots represent diversity measures (center line is median, lower, and upper hinges correspond to the first (Q1) and third (Q3) quartiles; the upper whisker is located at the smaller of the maximum alpha diversity measures and Q3 + 1.5 × IQR (Q3 − Q1); the lower whisker is located at the larger of the minimum alpha diversity measures and Q1 − 1.5 × IQR). Comparison is done using ANOVA * *p*-value < 0.05; ** *p*-value = 0.01–0.001; *** *p*-value < 0.001. Supplementary Figure S18: Beta diversity results: principal coordinates analysis (PCoA) of Bray–Curtis distances with respect to the three groups of body mass index class (BMIclass): normal, over (i.e., overweight) and obese. Each point represents one sample. Each color indicates sample metadata of BMI class.
