# Supplementary figures and images for: Gut Microbiota in Military International Travelers with Doxycycline Malaria Prophylaxis: Towards the Risk of a Simpson Paradox in the Human Microbiome Field
Source: Pathogens. 2021 Aug 21;10(8):1063. doi: 10.3390/pathogens10081063 (PMC8400693; doi:10.3390/pathogens10081063)

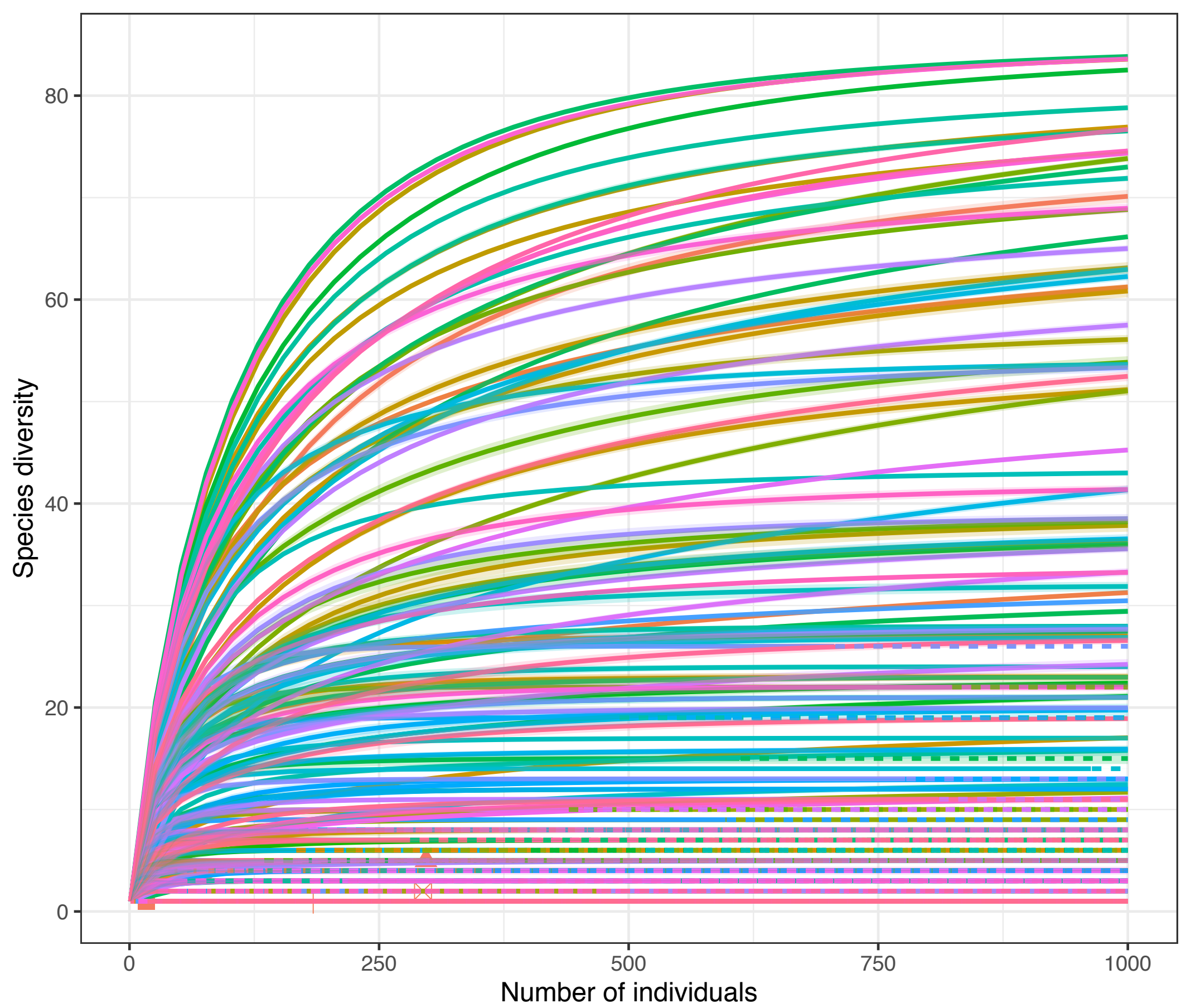

Supplement: Supplementary file 1 [file pathogens-10-01063-s001.zip › Suppl Fig S1 coverage.pdf]

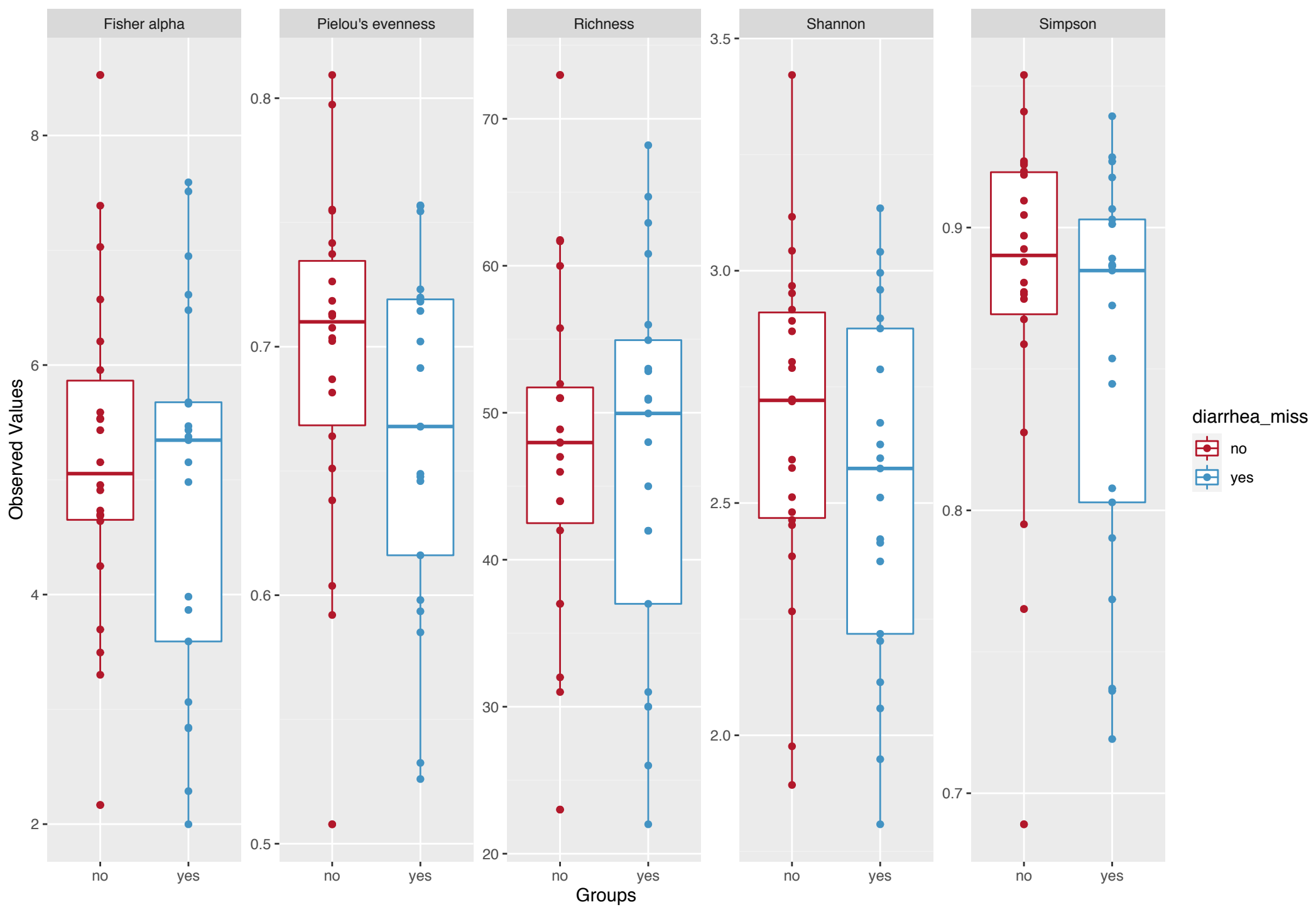

Supplement: Supplementary file 1 [file pathogens-10-01063-s001.zip › Suppl Fig S10 alphaD Diarrhea yes no_before.pdf]

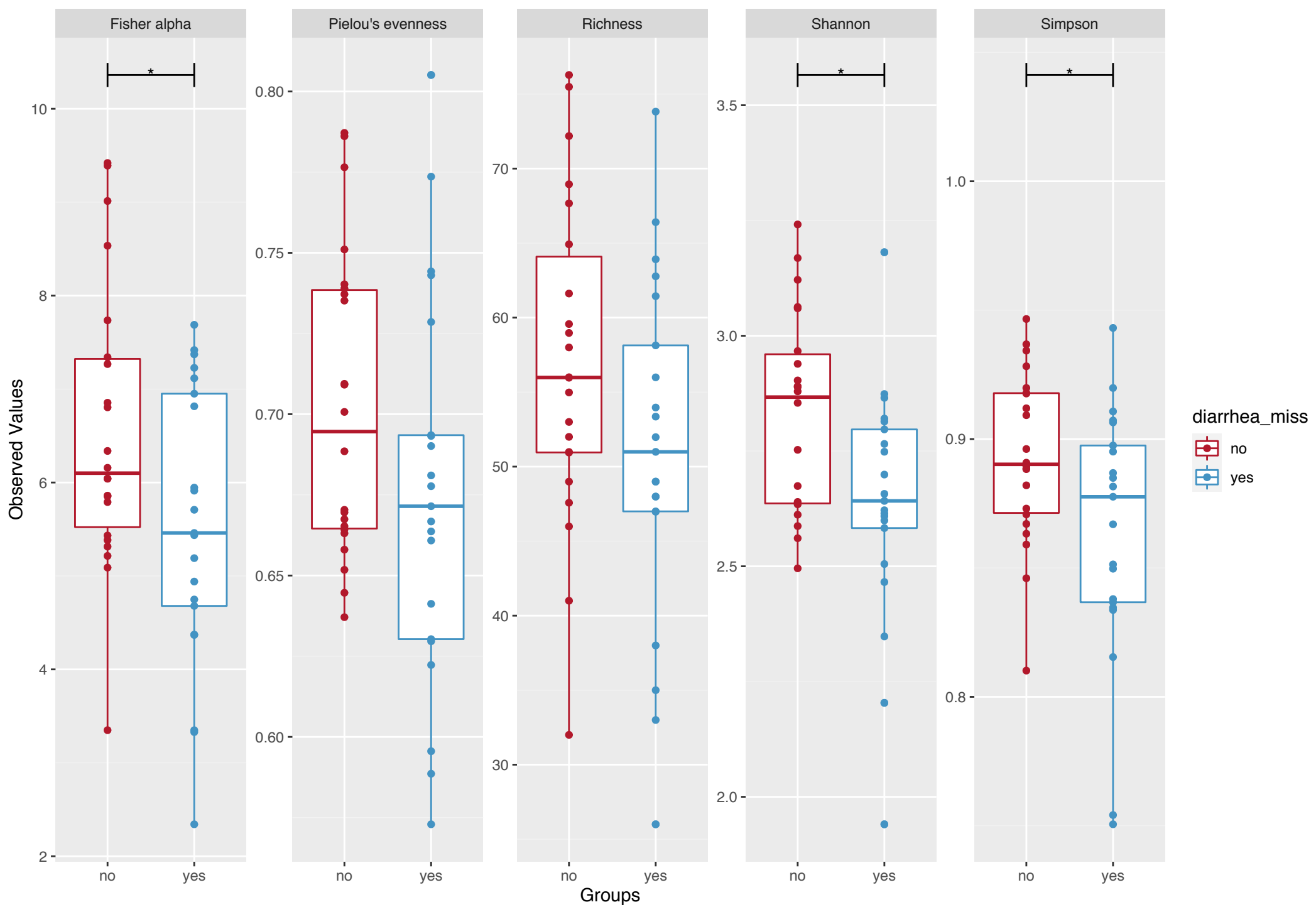

Supplement: Supplementary file 1 [file pathogens-10-01063-s001.zip › Suppl Fig S11 alphaD Diarrhea yes no_after.pdf]

Figure 2 : PCoA of Bray-Curtis distance

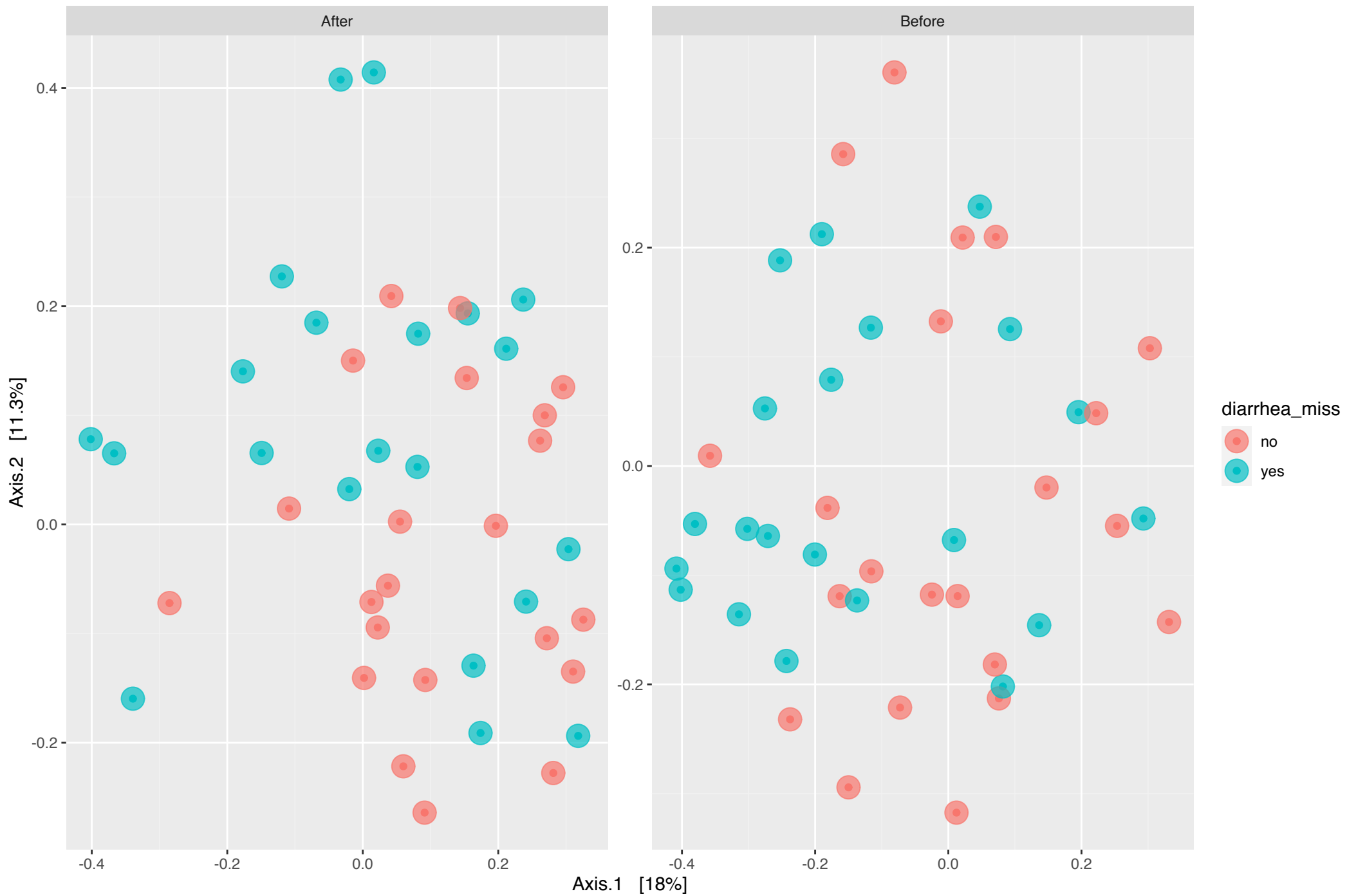

Supplement: Supplementary file 1 [file pathogens-10-01063-s001.zip › Suppl Fig S12 PCOA BC diarrhée yes no B and A.pdf]

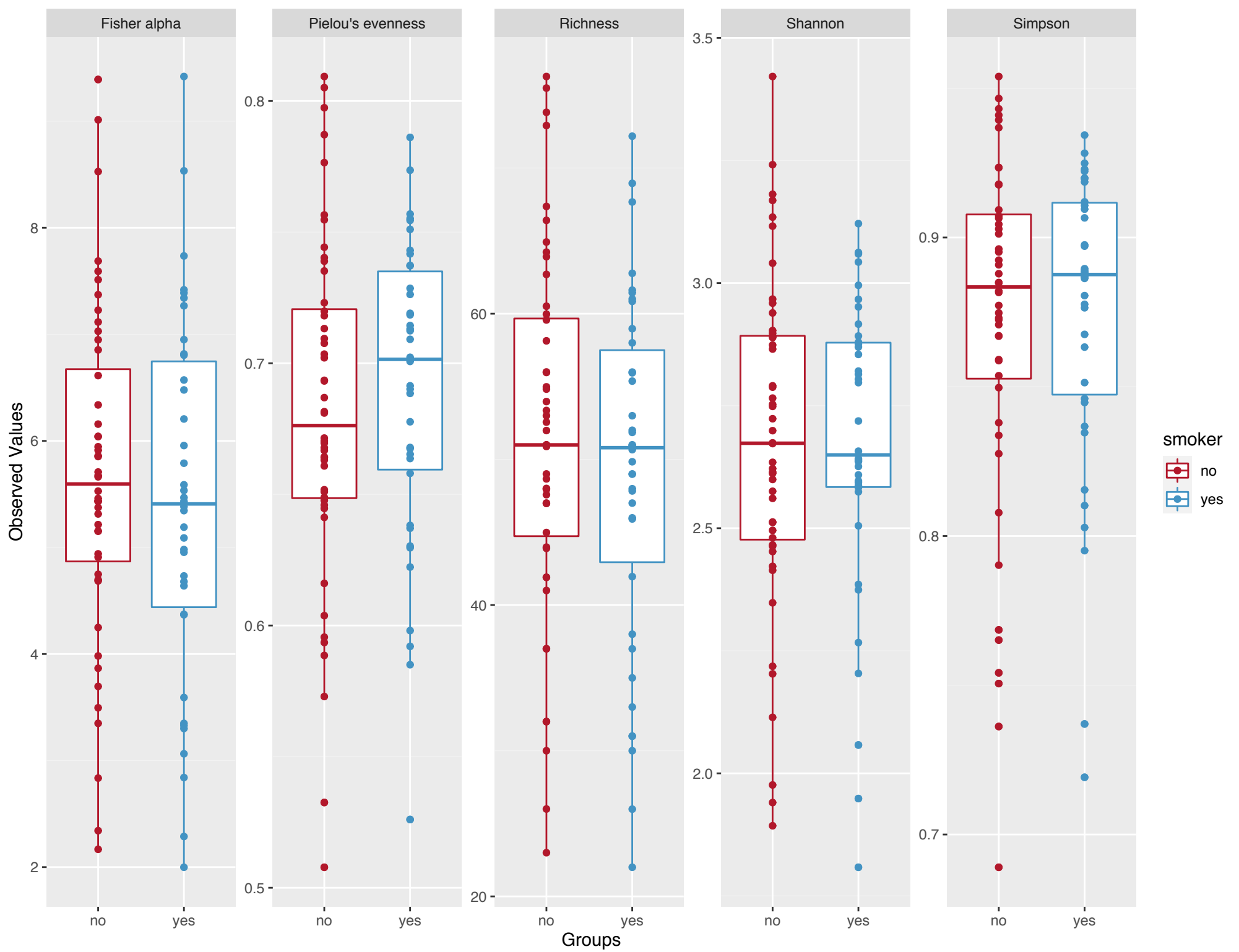

Supplement: Supplementary file 1 [file pathogens-10-01063-s001.zip › Suppl Fig S13 alpha D smoking.pdf]

Figure 2 : PCoA of Bray-Curtis distance

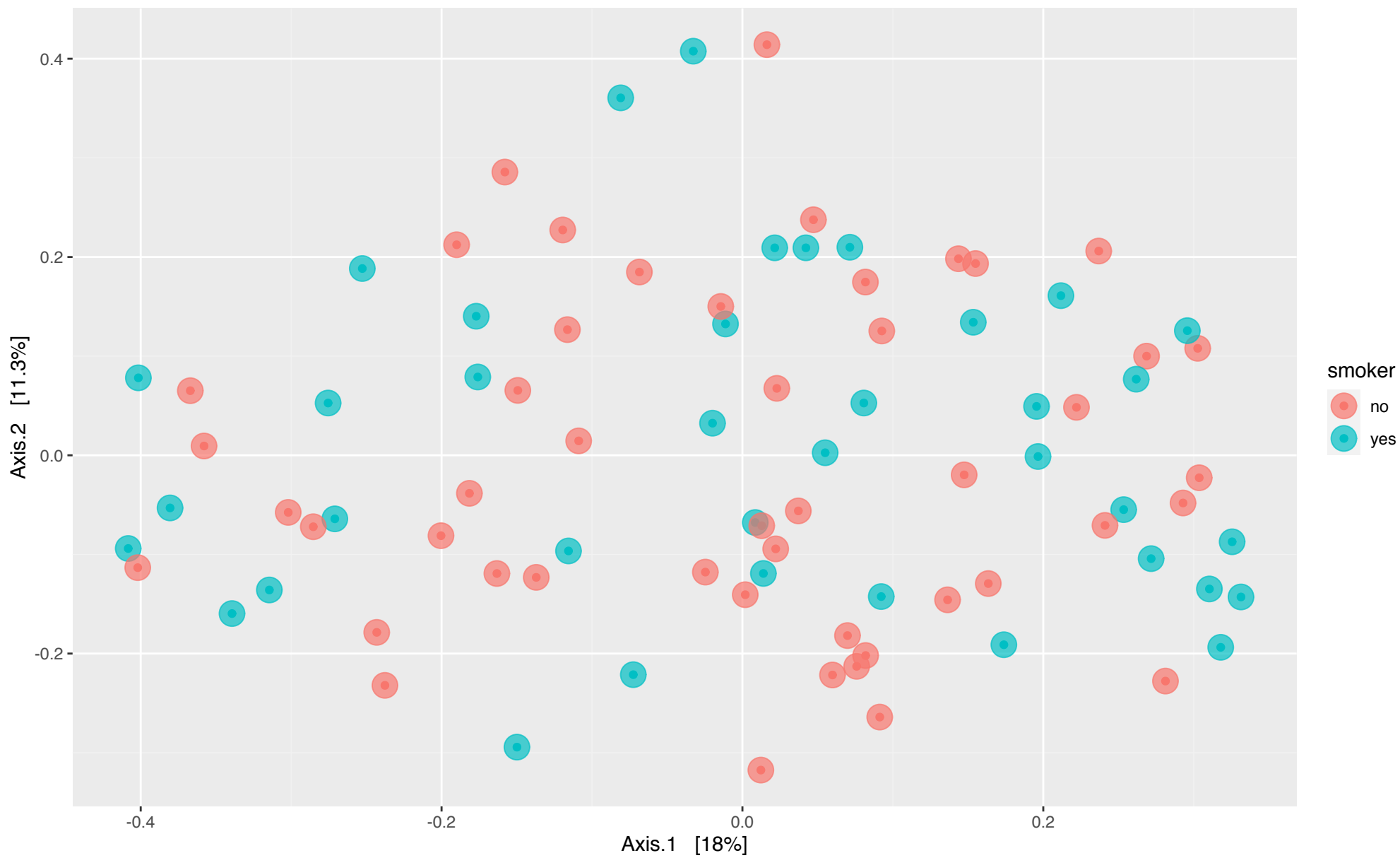

Supplement: Supplementary file 1 [file pathogens-10-01063-s001.zip › Suppl Fig S14 PCOA BC smoker.pdf]

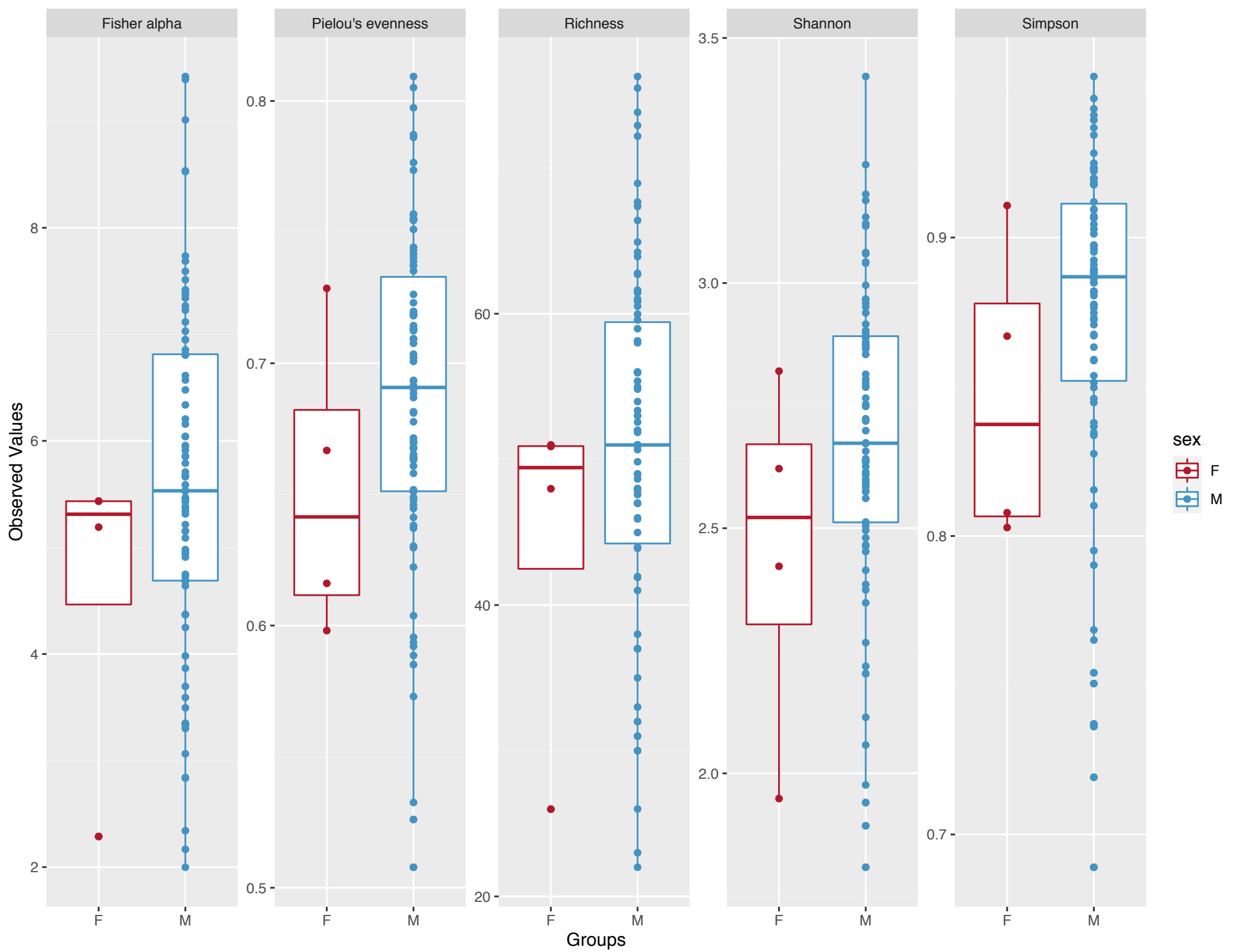

Supplement: Supplementary file 1 [file pathogens-10-01063-s001.zip › Suppl Fig S15 Alpha Sexe.pdf]

Figure 2 : PCoA of Bray-Curtis distance

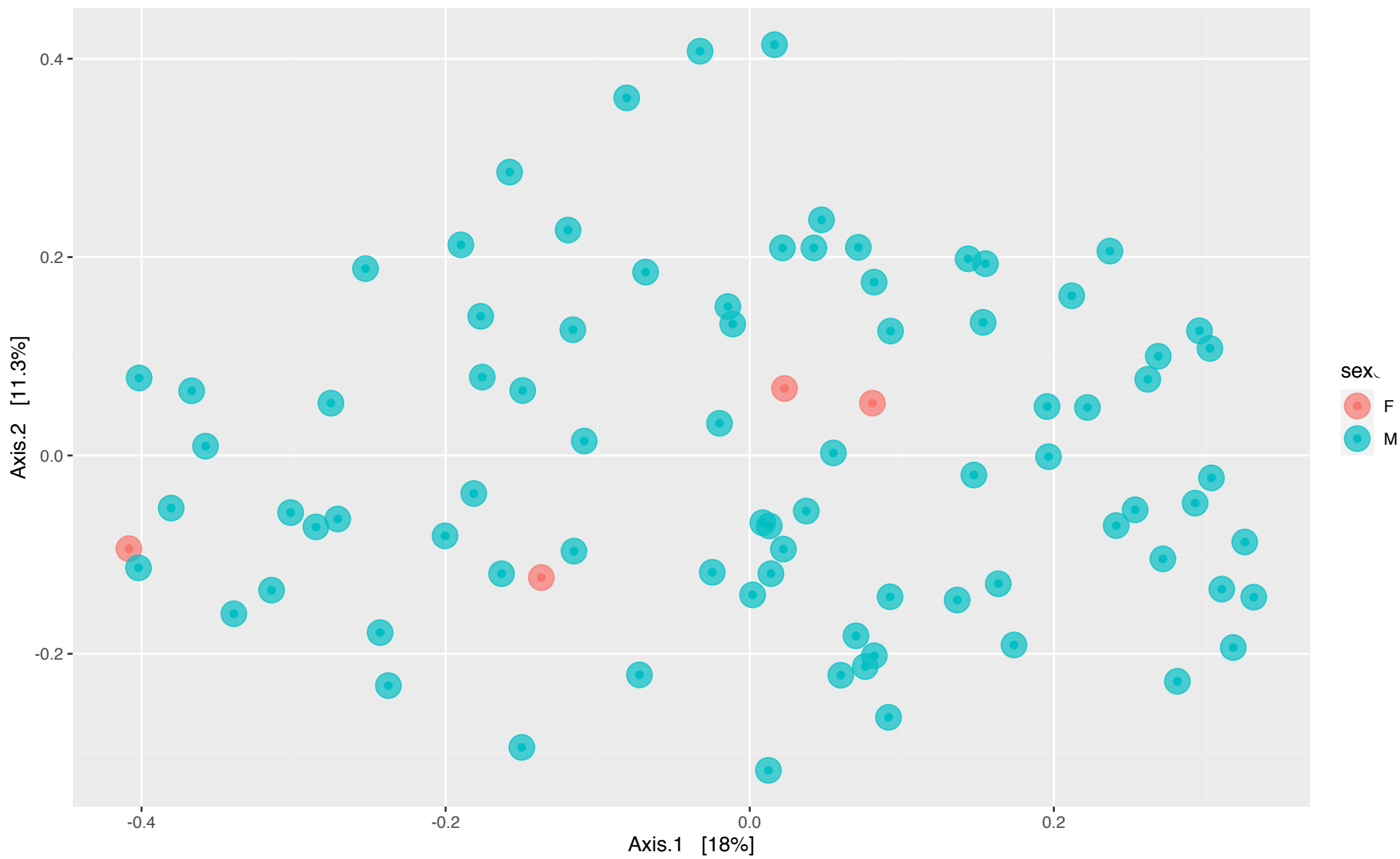

Supplement: Supplementary file 1 [file pathogens-10-01063-s001.zip › Suppl Fig S16 PCOA BC sex.pdf]

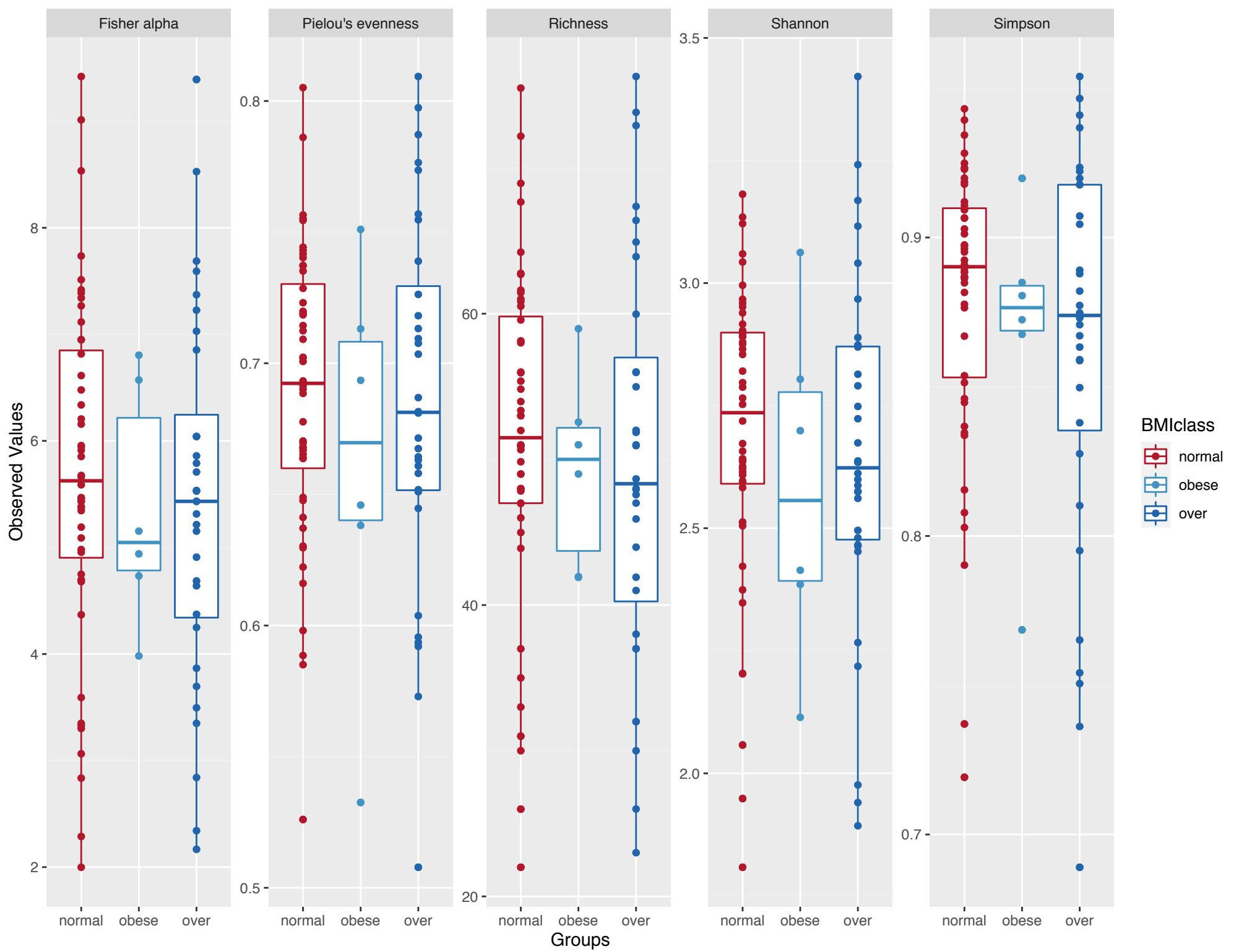

Supplement: Supplementary file 1 [file pathogens-10-01063-s001.zip › Suppl Fig S17 Alpha BMIClasss.pdf]

Figure 2 : PCoA of Bray-Curtis distance

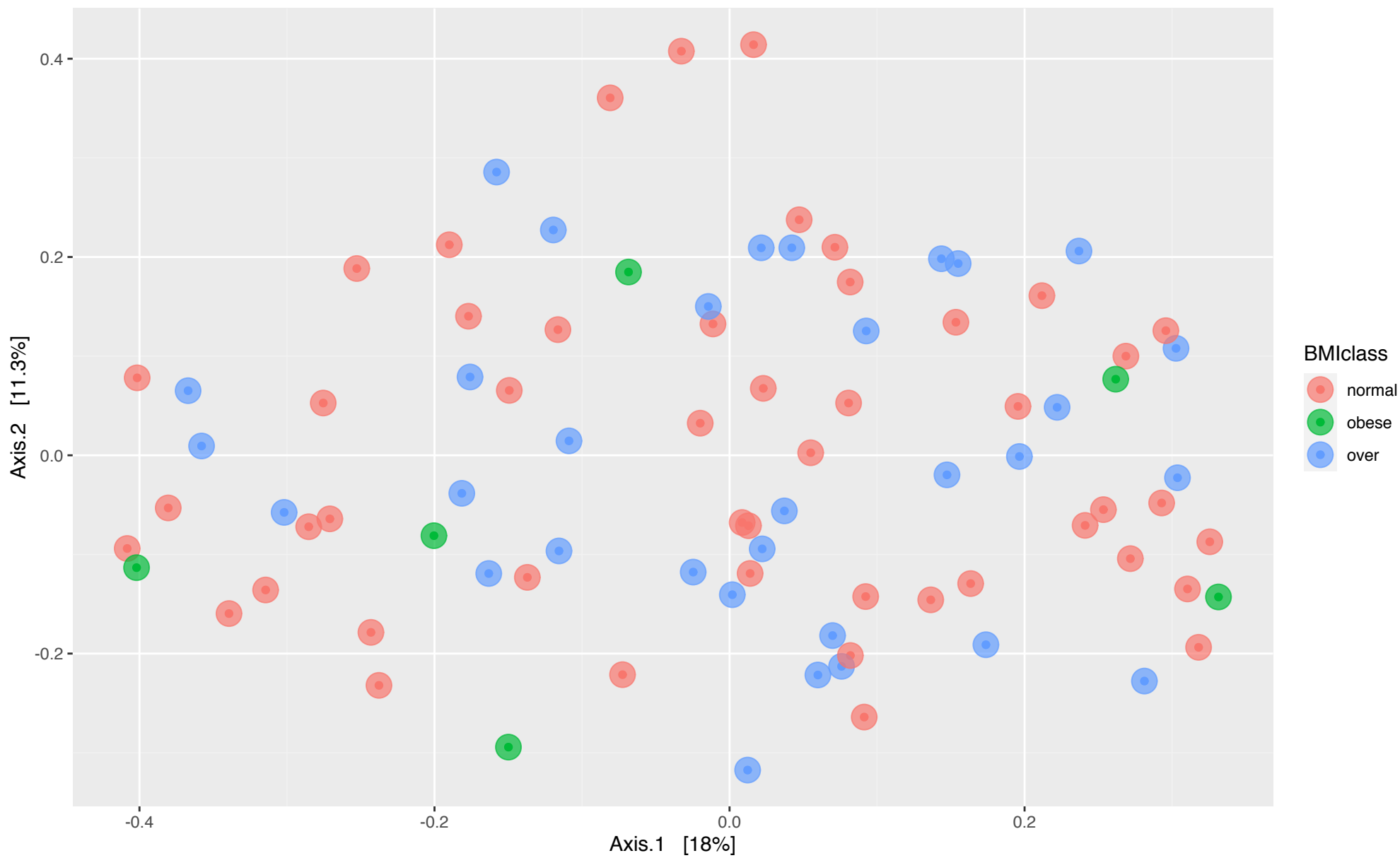

Supplement: Supplementary file 1 [file pathogens-10-01063-s001.zip › Suppl Fig S18 PCOA BC BMI class.pdf]

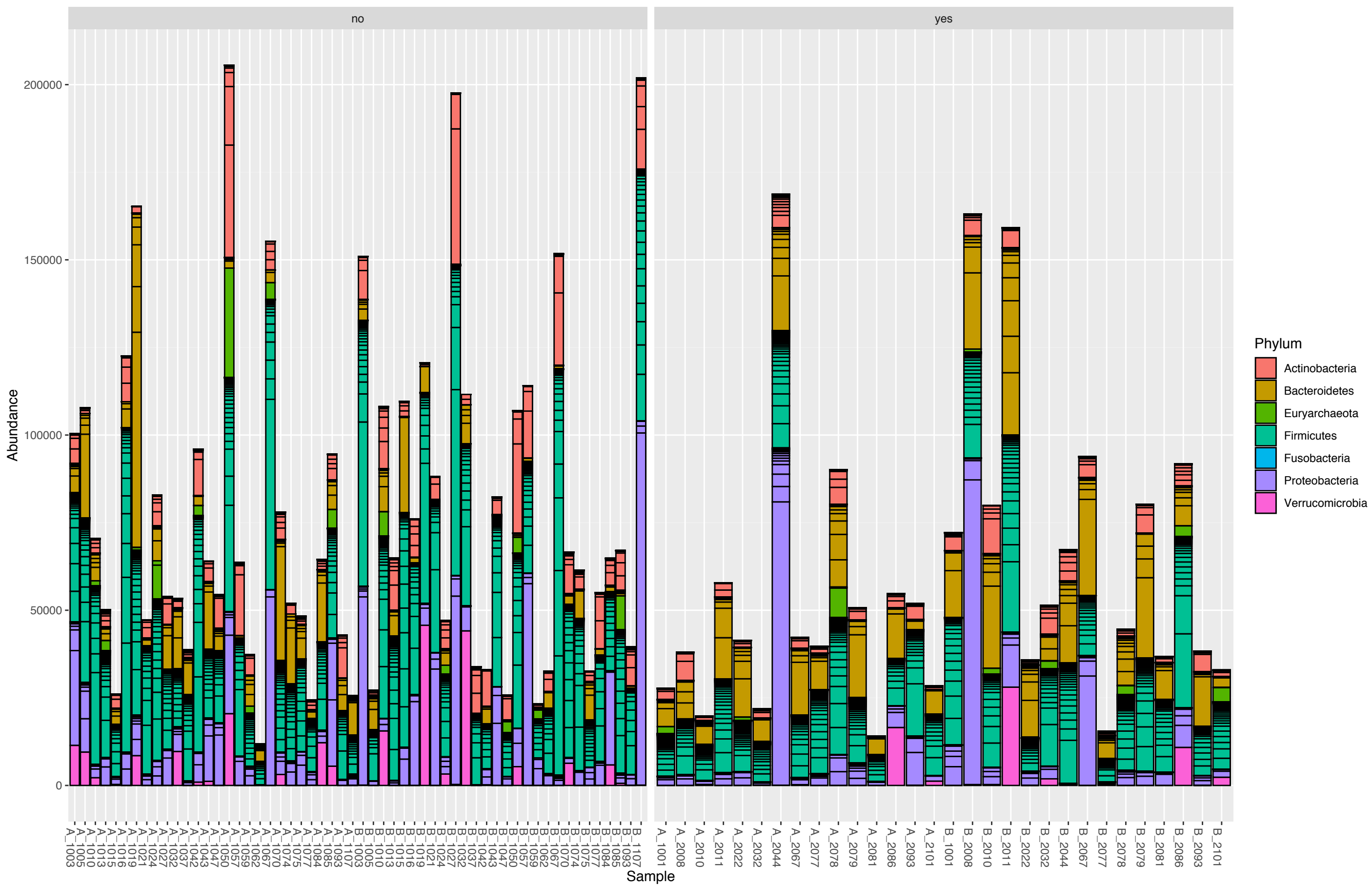

Supplement: Supplementary file 1 [file pathogens-10-01063-s001.zip › Suppl Fig S2 absolu abundance PHYLA doxy yes no B A.pdf]

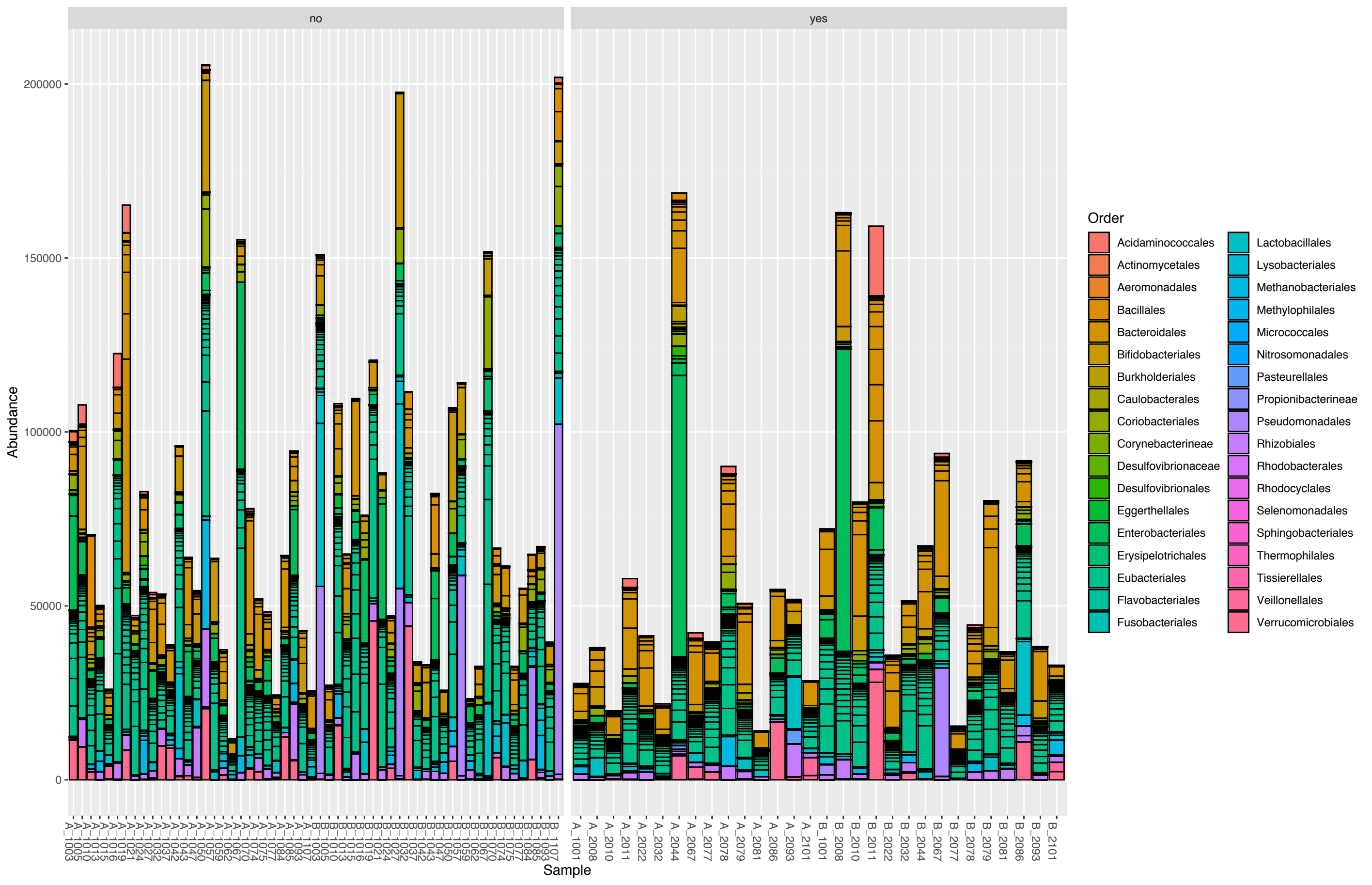

Supplement: Supplementary file 1 [file pathogens-10-01063-s001.zip › Suppl Fig S3 absolute adundance ORDER doxy yes no B A.pdf]

Composition within Firmicutes ( 15 top Genus )

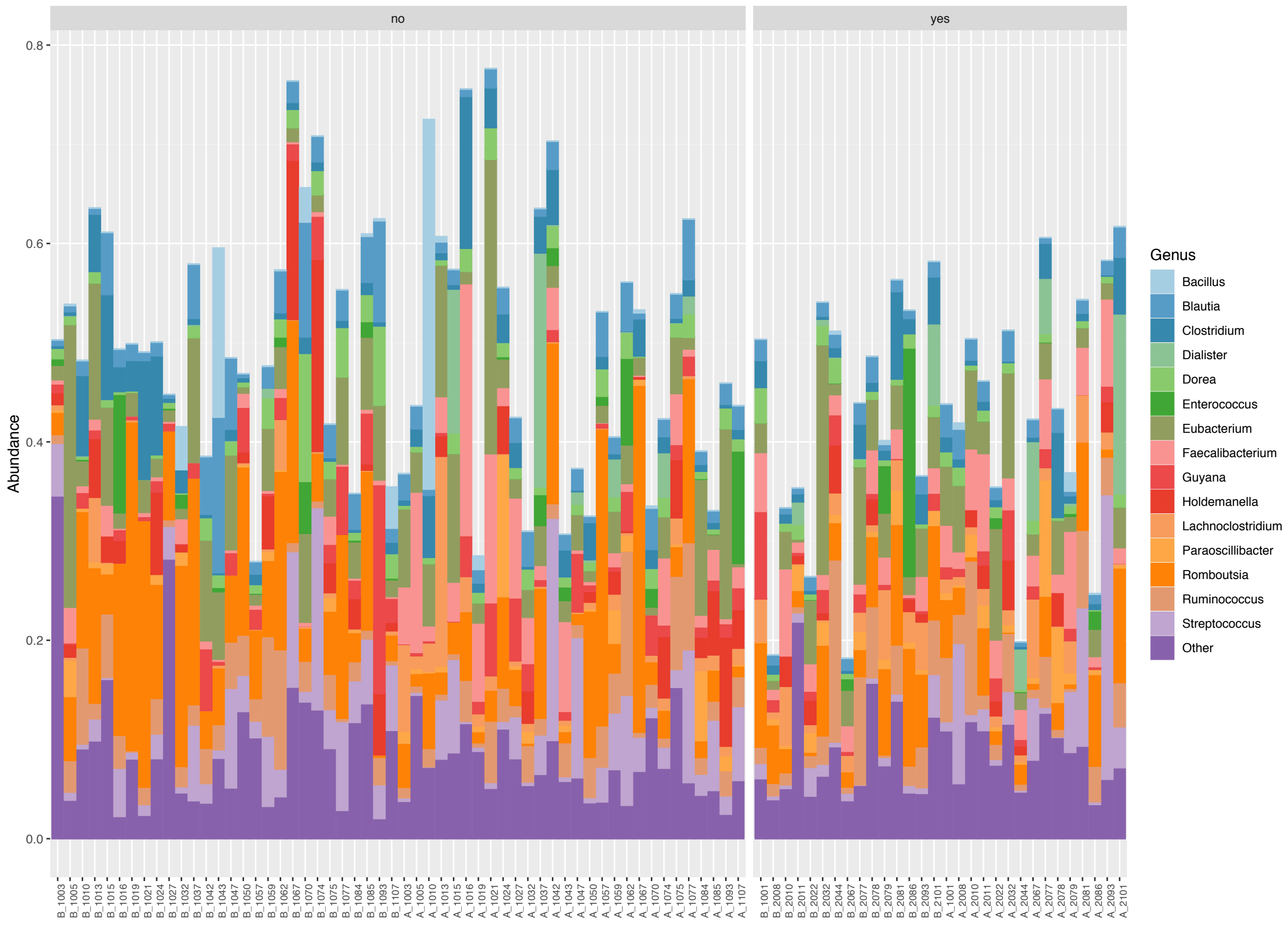

Supplement: Supplementary file 1 [file pathogens-10-01063-s001.zip › Suppl Fig S4 Firmicutes TOP 15_genus_doxy1.pdf]

Composition within Bacteroidetes ( 15 top Genus )

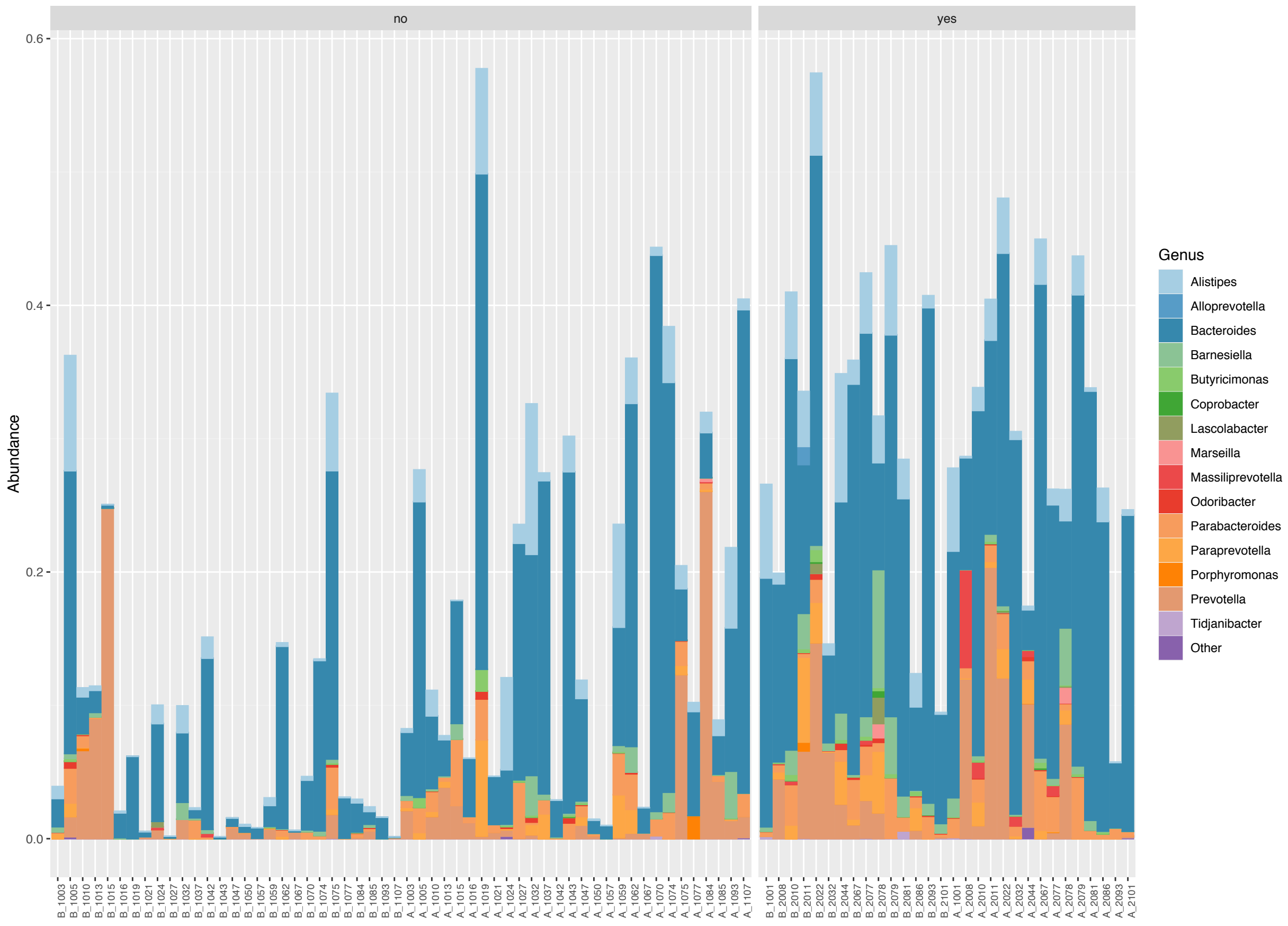

Supplement: Supplementary file 1 [file pathogens-10-01063-s001.zip › Suppl Fig S5 Bacteroidetes TOP 15_genus_doxy1.pdf]

Composition within Proteobacteria ( 15 top Genus )

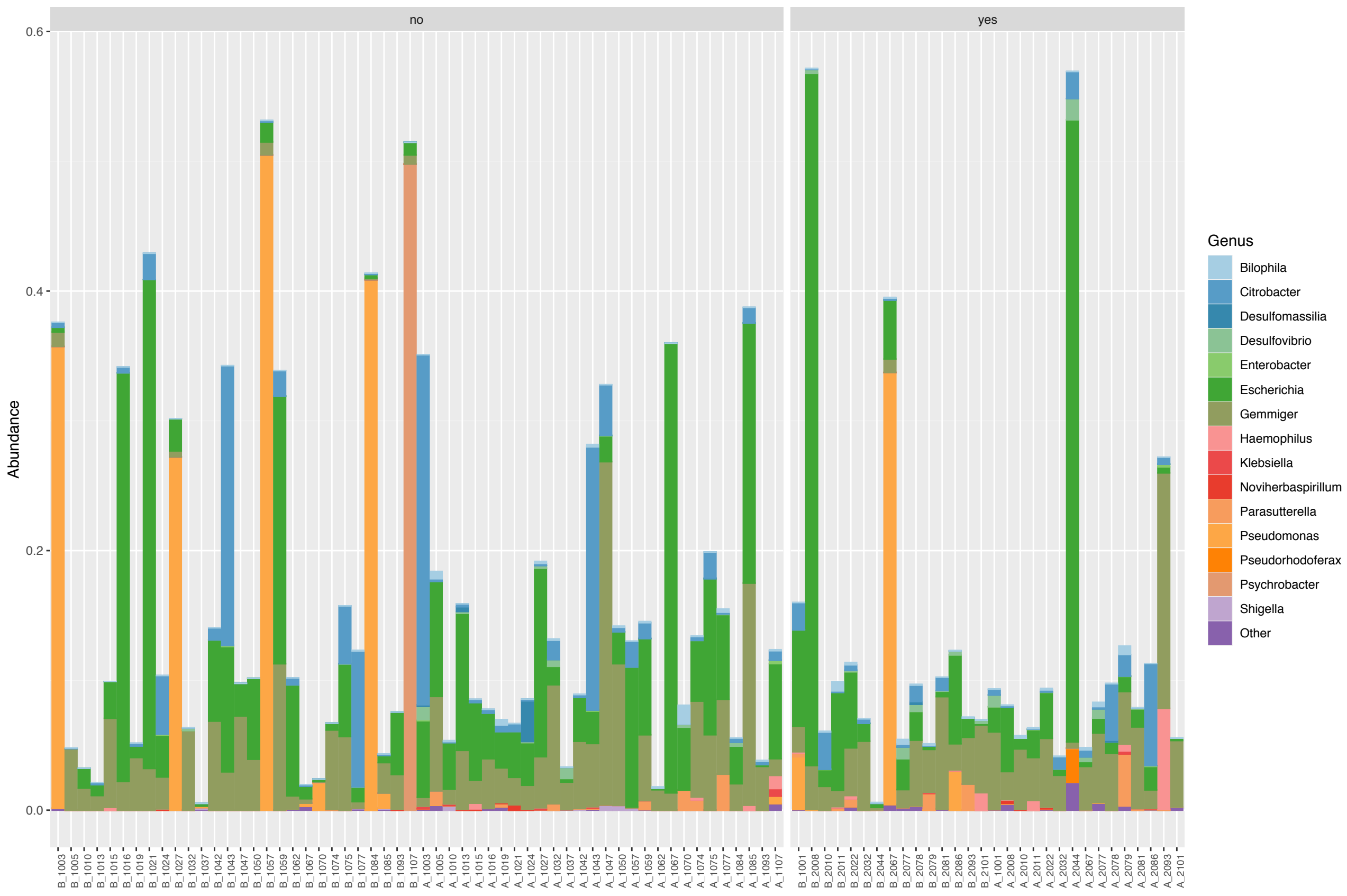

Supplement: Supplementary file 1 [file pathogens-10-01063-s001.zip › Suppl Fig S6 Proteobacteria TOP 15_genus_doxy1.pdf]

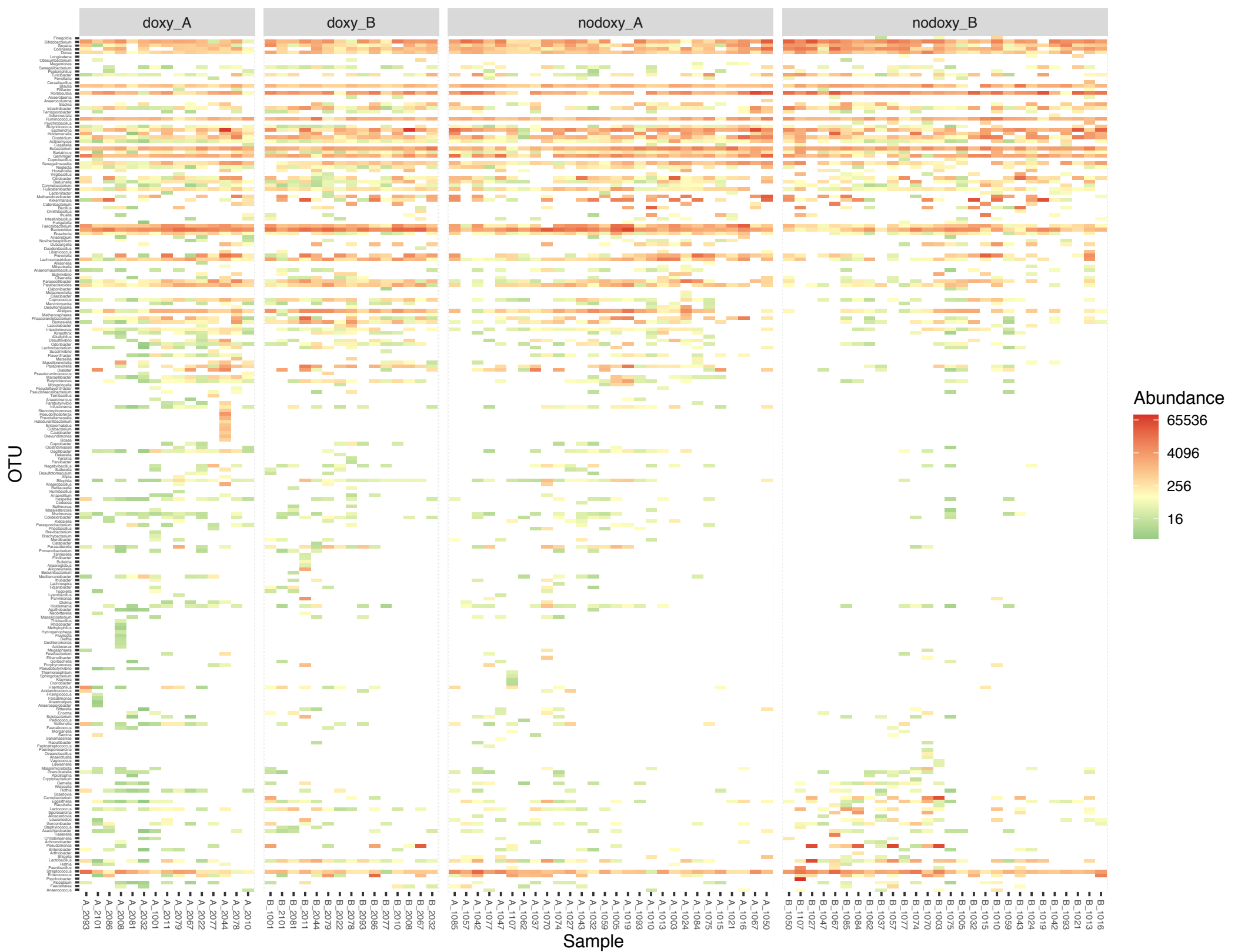

Supplement: Supplementary file 1 [file pathogens-10-01063-s001.zip › Suppl Fig S7 4 HM Abondancedoxy2.pdf]

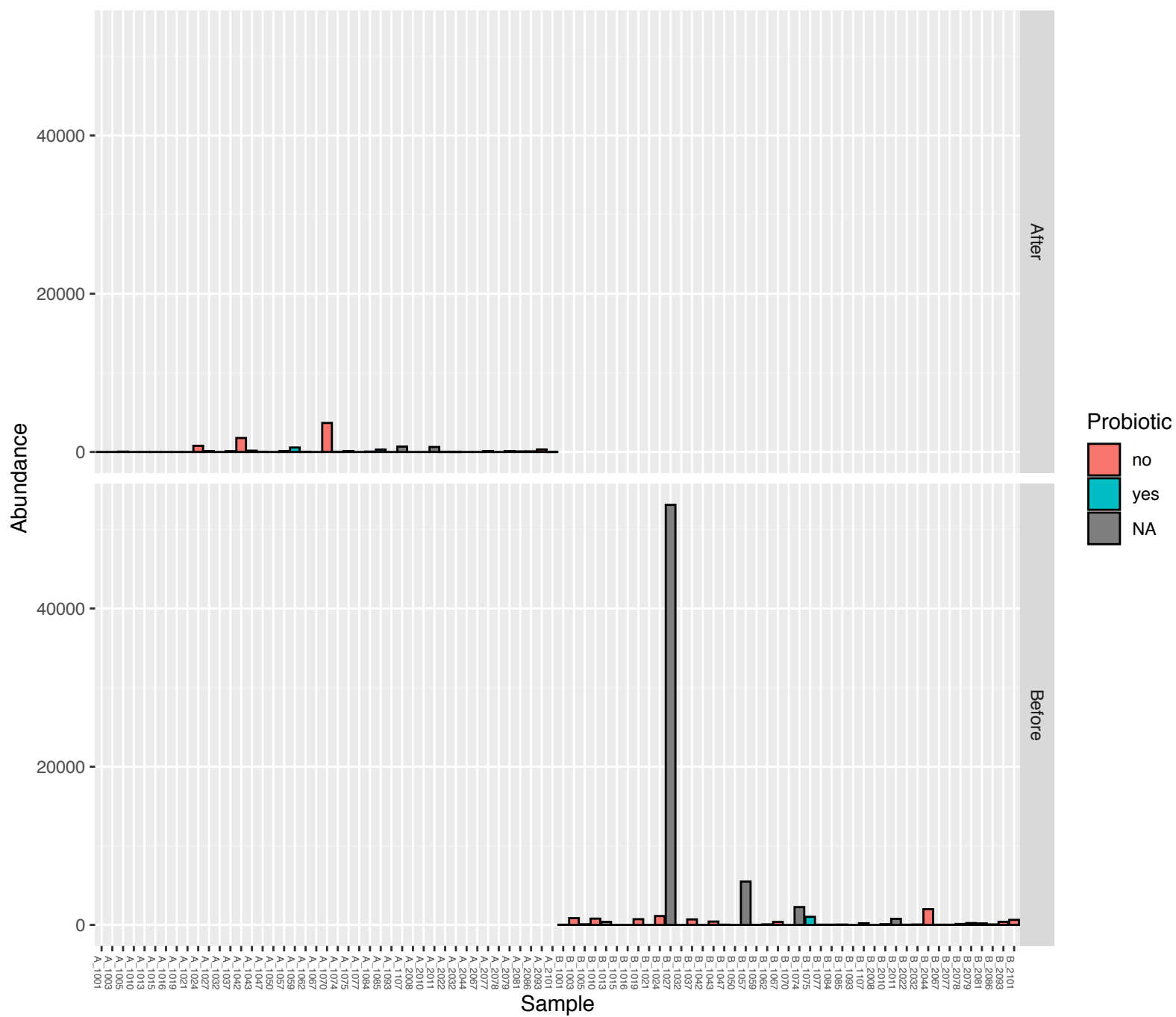

Supplement: Supplementary file 1 [file pathogens-10-01063-s001.zip › Suppl Fig S8a Lactobacillus abundance per sample B A and probiotics satuts.pdf]

# Lactobacillus only

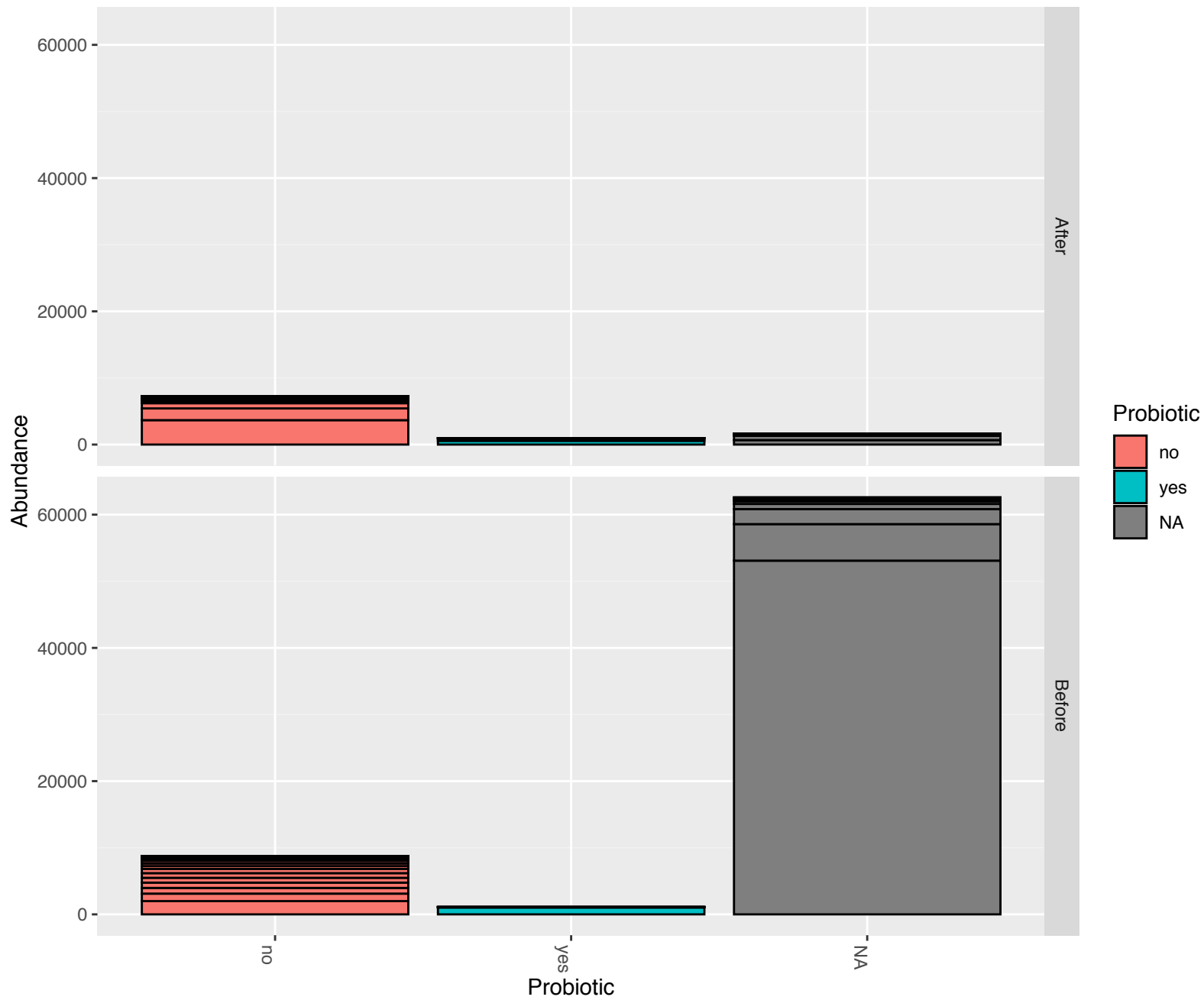

Supplement: Supplementary file 1 [file pathogens-10-01063-s001.zip › Suppl Fig S8b Lactobacillus abundance per sample of probiotic groups B and A.pdf]

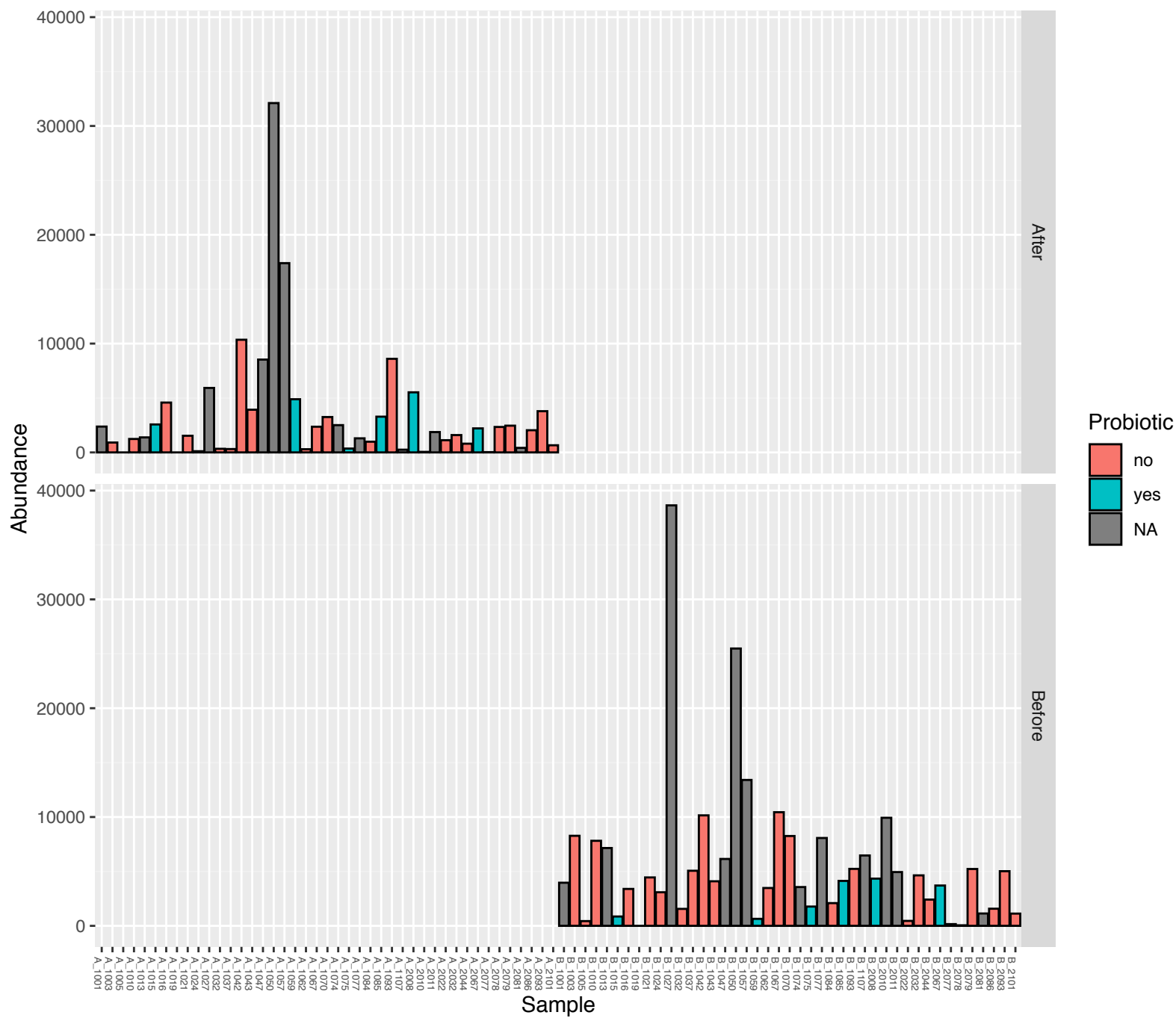

Supplement: Supplementary file 1 [file pathogens-10-01063-s001.zip › Suppl Fig S9 a Bifidobacterium abundance per sample B A and probiotics satuts.pdf]

Bifidobacterium only by Probiotic status

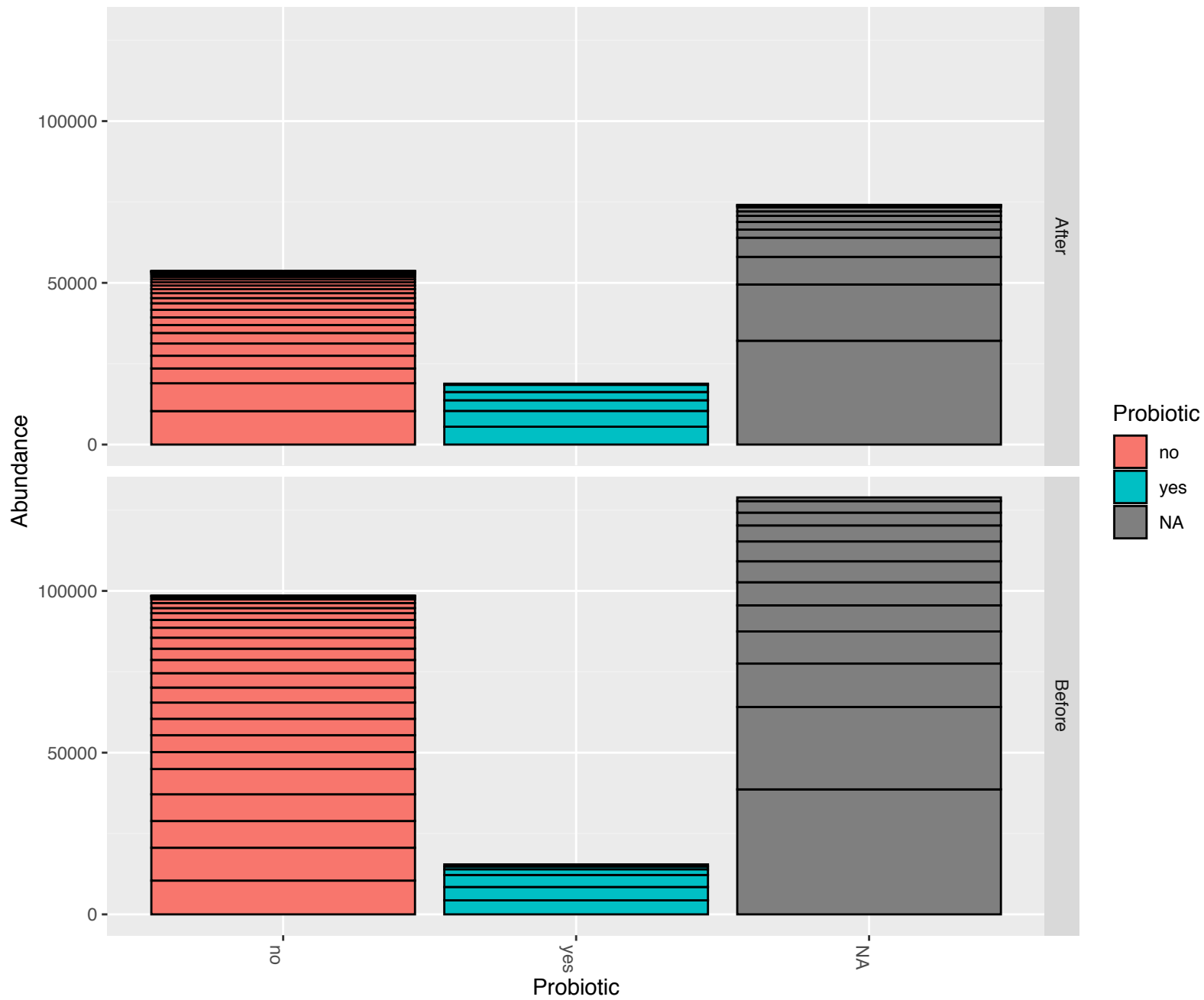

Supplement: Supplementary file 1 [file pathogens-10-01063-s001.zip › Suppl Fig S9 b Rplot Bifidobacterium abundance per sample of probiotic groups B and A.pdf]
